# Environmental DNA Metabarcoding Reveals Spatiotemporal Variation in Fish Communities of the Middle Raohe River, China

**DOI:** 10.3390/biology15201812

**Published:** 2026-10-10

**Authors:** Qin Ma, Tonglin Zhang, Min Zeng, Qi Hua, Sheng Wang

**Affiliations:** 1 School of Life Sciences, Nanchang Normal University, Nanchang 330032, China; 2 Aquatic Conservation and Rescue Center of Jiangxi Province, Nanchang 330029, China

**Keywords:** community composition, freshwater biodiversity, molecular monitoring, paired-site surveys, Raohe River

## Abstract

Environmental DNA (eDNA) metabarcoding can provide fish-community inventories across river networks. We surveyed ten sites in the Jingdezhen section of the Raohe River system on 20–23 October 2023 and 2–5 June 2024, covering the Donghe River, Chang River, and Le’an River. Three technical process replicates were pooled per site and campaign to produce 20 community profiles. The two surveys detected 96 and 94 taxa, respectively, including 100 fish taxa overall, of which 90 were shared. Richness, Shannon diversity, Simpson diversity, and Pielou evenness did not differ significantly between paired campaigns. Community composition differed between campaigns (Bray–Curtis PERMANOVA: R^2^ = 0.293; Jaccard: R^2^ = 0.185; both adjusted *p* = 0.002). PERMDISP did not detect unequal dispersion. River-group differentiation was clearer in 2023, whereas neither distance metric was significant in 2024 after multiple-testing correction. A reduced environmental partial RDA accounting for site and campaign effects was not significant (F = 0.971, *p* = 0.420). These findings provide a two-campaign molecular inventory for the ten surveyed sites and identify between-campaign compositional differences, without demonstrating seasonal dynamics or independent environmental drivers.

## Simple Summary

Reliable information on fish diversity is needed to guide river monitoring, but conventional capture surveys can miss some species. We analyzed traces of fish DNA in river water from ten sites in the Jingdezhen section of the Raohe River system during two campaigns. We detected 100 fish taxa overall. No significant differences in local diversity measures were detected between campaigns, whereas the composition of DNA detections differed. Differences among the three river groups were clearer in 2023 than in 2024. These results provide a molecular baseline for the surveyed sites and show how water-DNA analysis can complement conventional fish surveys.

## 1. Introduction

Freshwaters support exceptional biodiversity and provide essential services [1,2]. Yet flow regulation, habitat fragmentation, pollution, invasive species, and overexploitation threaten their biota [3,4,5]. A global assessment classified 24% of assessed freshwater fishes, decapod crustaceans, and odonates as threatened with extinction [6]. Conservation responses call for spatially explicit biodiversity information alongside action on flows, water quality, and habitat [7,8]. Fish assemblages integrate pressures across habitats and trophic levels, making them useful in ecological monitoring. Representative inventories remain difficult to obtain across large, heterogeneous river networks because taxa differ in habitat use, body size, behavior, and catchability.

Conventional netting and electrofishing remain essential because they provide voucher-based identifications and, when designed appropriately, information on body size, numerical abundance, and biomass. However, each gear samples only part of a heterogeneous fish assemblage and can be selective with respect to species, body size, and habitat; capture surveys can also be labor-intensive and disturb organisms and habitats [9,10,11]. These constraints motivate complementary approaches that can broaden taxonomic and spatial coverage, especially in networks containing connected mainstem, tributary, and lentic-influenced habitats.

Environmental DNA metabarcoding addresses part of this need by detecting DNA released by organisms into water and assigning high-throughput sequence reads to taxa using a reference database. Fish-specific mitochondrial 12S markers can recover diverse assemblages from relatively small water samples, often detecting taxa missed during an individual capture survey [9,10,11,12]. Its performance depends on sampling design, primer choice, reference sequences, and bioinformatic filtering; incomplete reference libraries and methodological variation can affect taxonomic recovery [13]. Accordingly, eDNA is best framed as a complementary community-assessment approach whose results should be interpreted alongside field design and, where available, conventional surveys.

Riverine eDNA studies show that molecular samples can retain ecologically meaningful among-reach community patterns despite downstream DNA movement [10,11,14,15]. In China, eDNA metabarcoding has documented fish-community variation in species-rich river systems [16,17]. Because an eDNA signal is transported in flowing water, its spatial interpretation should be considered in relation to river connectivity and field design [10,14].

The Raohe River is an important tributary system connected to Poyang Lake in Jiangxi Province, China, and comprises the Chang River and the Le’an River as its two principal components. Conventional fish surveys have provided valuable but spatially and temporally heterogeneous information from parts of this system. A July 2023 survey of the middle Chang River recorded 53 fish species from four orders and nine families, with Cypriniformes accounting for 69.81% of the inventory [18]. A survey of the Fenghuang Bridge reach in April 2025 recorded 38 species, whereas a 2023 investigation of Wuxikou Reservoir recorded 40 species [19,20]. These studies characterize particular river reaches or reservoir habitats, but differences in location, habitat, sampling date, gear, and effort preclude direct comparison. A molecular assessment of fish communities across the middle Raohe River has remained limited. The connected tributary, river-channel, and reservoir-influenced habitats of the middle Raohe provide a relevant setting for examining how fish detections vary within a river network draining to Poyang Lake.

Here, we applied eDNA metabarcoding in two survey periods to establish a molecular inventory of fish communities in the middle Raohe River. We aimed to (1) characterize the fish taxa detected in the two survey periods; (2) describe site-level richness, Shannon diversity, Simpson diversity, and Pielou evenness; (3) evaluate temporal and river-group differences in community composition using abundance-weighted and presence–absence dissimilarities; and (4) examine associations between fish-community composition and concurrently measured environmental variables.

## 2. Materials and Methods

### 2.1. Study Area and Sampling Design

**Figure 1:**
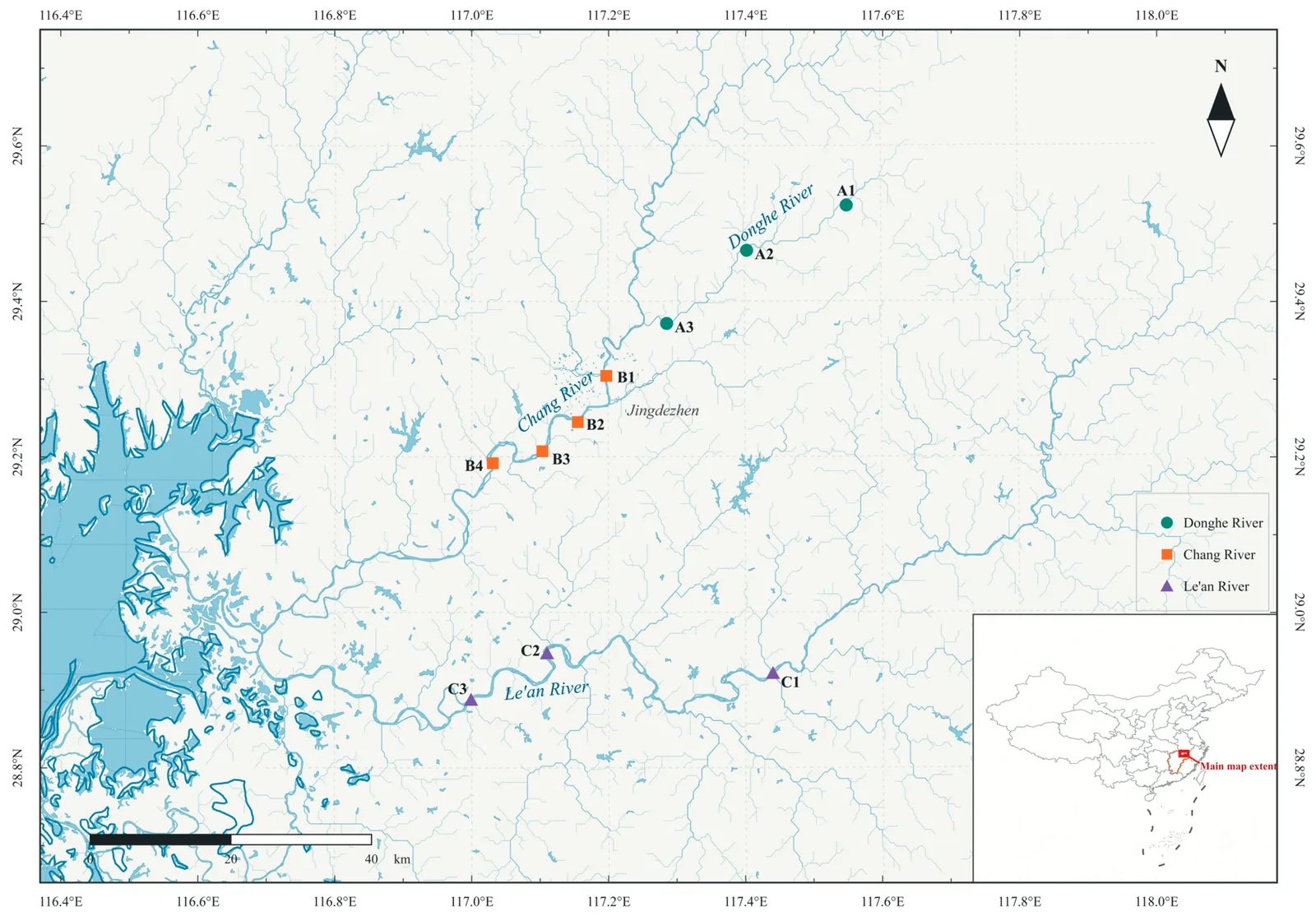
Sampling sites in the middle Raohe River system within Jingdezhen, China: Donghe River (A1–A3), Chang River (B1–B4), and Le’an River (C1–C3). The red box in the China locator inset marks the geographic extent displayed in the main map.

The study covered surveyed middle reaches of the Raohe River system within Jingdezhen, Jiangxi Province, China, on 20–23 October 2023 and 2–5 June 2024 (Figure 1). This study area did not represent the full Raohe River basin. The sampling design comprised three connected river groups: the Donghe River (A1–A3), Chang River (B1–B4), and Le’an River (C1–C3). The Donghe River is a tributary of the Chang River; the Chang and Le’an rivers are the two principal components of the Raohe River system. Sites sampled on the same day were visited from upstream to downstream. Coordinates and straight-line intersite distances are provided in Table S8; these distances do not represent channel travel distances.

At each site, water was collected from three nearby points and three vertical layers: the surface, approximately 1 m below the surface, and a bottom layer at least 2 m above the sediment. One liter was collected from each point–depth combination, yielding a 9 L composite site sample. Three 500 mL subsamples were independently filtered, extracted, and amplified. Because these subsamples originated from one composite sample, they were technical process replicates, not independent biological replicates. Replicate read profiles were summed to produce 20 site-by-campaign units, representing ten repeatedly sampled sites.

### 2.2. eDNA Filtration, Extraction, and PCR Amplification

Water samples were kept chilled and filtered within 1–2 h of collection using a solvent-filtration vacuum pump assembly (Tianjin Jinteng Experimental Equipment Co., Ltd., Tianjin, China). Each 500 mL subsample was filtered through one glass-fiber membrane with a pore size of 0.45 µm and a diameter of 60 mm (Shanghai Xingya Purification Materials Factory, Shanghai, China). Membranes were placed in tubes and stored at −80 °C before DNA extraction, which took place within 24 h of freezing. DNA was extracted using the PowerWater DNA Isolation Kit (MO BIO Laboratories, Carlsbad, CA, USA), following the manufacturer’s protocol. One field blank per site was carried through filtration, extraction, PCR, and gel screening. One additional PCR-negative control, with sterile water replacing the DNA template, was included per batch of 30 technical process samples. No visible target bands were observed in the controls, which were not sequenced. No positive control or mock community was included.

A short mitochondrial 12S rRNA fragment was amplified using Tele02F (5′-AAACTCGTGCCAGCCACC-3′) and Tele02R (5′-GGGTATCTAATCCCAGTTTG-3′) primers [9]. PCR was performed on an ABI GeneAmp 9700 thermocycler (Applied Biosystems, Carlsbad, CA, USA) using TransStart FastPfu DNA Polymerase (AP221-02; TransGen Biotech, Beijing, China). Each 20 µL reaction contained 4 µL of 5× FastPfu buffer, 2 µL of 2.5 mM dNTPs, 0.8 µL of each primer (5 µM), 0.4 µL of polymerase, 10 ng of template DNA, and nuclease-free water to 20 µL. The thermal program comprised 95 °C for 5 min, 35 cycles of 95 °C for 30 s, 58 °C for 30 s and 72 °C for 45 s, followed by 72 °C for 10 min and holding at 10 °C.

Amplicons were examined on 2% agarose gels stained with GelRed (Biotium, Fremont, CA, USA), using a DL2000 DNA Marker (Takara Biomedical Technology (Beijing) Co., Ltd., Beijing, China) and an FR-980A gel imaging system (Furi Technology, Shanghai, China). Products were gel-purified using the AxyPrep DNA Gel Extraction Kit (Axygen, Union City, CA, USA). Gel electrophoresis and excision were performed in a dedicated post-PCR area with unidirectional workflow. Separate disposable blades and clean work-surface coverings were used for individual samples, gloves were changed between handling steps, and excised products were transferred to separate labeled tubes. Purified products were pooled and sequenced by Shanghai BIOZERON Biotechnology Co., Ltd. (Shanghai, China) using an Illumina MiSeq system (Illumina, Inc., San Diego, CA, USA) with paired-end 250-bp sequencing.

### 2.3. Bioinformatic Processing and Taxonomic Assignment

Demultiplexed reads were processed using DADA2 version 1.18.0 for quality filtering, error correction, sequence inference, and chimera removal [21]. Representative sequences were initially annotated using BLASTn version 2.2.30 with an E-value threshold of 10^−10^. Reference sequences comprised freshwater fish 12S rRNA and mitochondrial genome sequences from the NCBI nucleotide database. Fish taxonomic assignments were reviewed against historical fish inventories and published occurrence records for the Poyang Lake basin to screen for biogeographically implausible identifications [18,19,20,22]. Scientific names, taxonomic classification, and nomenclatural authorities were checked primarily against the Species 2000 China catalogue and Species Diversity and Distribution of Inland Fishes in China [23,24], with FishBase and Eschmeyer’s Catalog of Fishes consulted where these sources did not provide the required information [25,26]. Genus- and species-level nomenclatural authorities are provided in Tables S1 and S2. Unclassified records and taxa with zero reads in the analyzed samples were excluded. The final fish-taxon count matrix was used for the primary analyses; additional low-read filtering was evaluated separately as a sensitivity analysis.

### 2.4. Technical-Replicate Quality Control

Read depth and detected richness were summarized for each technical process replicate. A replicate was flagged as low depth when its total read count was below 10% of the median for its campaign. Within-site, within-campaign Bray–Curtis dissimilarities documented technical variability (Table S6). All three replicates were retained in the pooled profile. To assess unequal weighting, relative read abundance (RRA) was also calculated separately for each replicate and averaged equally within each site–campaign unit. Low-read sensitivity analyses set counts of ≤1, ≤2, <5, ≤5, or ≤10 to zero within technical replicates before pooling, and separately applied <5, ≤5, or ≤10 thresholds after pooling (Table S7). These sensitivity scenarios did not replace the primary count matrix.

### 2.5. Alpha Diversity

Alpha diversity was calculated from pooled site–campaign counts using observed richness, Shannon diversity, Simpson diversity, and Pielou evenness. Pielou evenness was Shannon diversity divided by the natural logarithm of richness. Expected richness was computed analytically using rarefy in vegan at 872,923 reads, the minimum pooled depth; this calculation does not use random rarefaction iterations. Site-level rarefaction curves are shown in Figure S2.

Campaign differences were tested across ten paired sites. Shapiro–Wilk tests assessed the normality of paired differences. Paired *t*-tests were used for the five alpha-diversity measures, whose differences satisfied this check; mean paired differences and 95% confidence intervals were reported. River-group differences were assessed within each campaign using Kruskal–Wallis tests. Benjamini–Hochberg (BH) correction was applied separately to the five paired tests and the ten river-group tests (five measures × two campaigns). Paired *t*-tests were two-sided (Table S4).

### 2.6. Beta Diversity

Bray–Curtis dissimilarity was calculated from RRA and used as the primary abundance-weighted beta-diversity metric. Jaccard dissimilarity based on presence–absence data was used as a complementary sensitivity analysis. Bray–Curtis patterns were visualized using principal coordinates analysis (PCoA).

PERMANOVA was implemented using adonis2 in vegan. Campaign models used dissimilarity~campaign with permutations restricted within site, enumerating the 1023 nonidentity exchanges of the ten site pairs. River models used dissimilarity~river separately within each campaign, with 9999 unrestricted permutations of its ten sites. Dispersion was assessed using betadisper with group medians and permutest, using the corresponding permutation matrices. BH correction was applied separately to the two campaign tests and four river tests, with PERMANOVA and PERMDISP treated as distinct test families. Campaign R^2^ confidence intervals were estimated using 1999 paired-site cluster bootstrap samples. These intervals are approximate because only ten independent site clusters were available.

### 2.7. Environmental Variables and Constrained Ordination

Environmental sampling coincided with eDNA sampling. Water temperature, pH, and dissolved oxygen were measured using a portable YSI ProQuatro multiparameter analyzer (YSI Inc., Yellow Springs, OH, USA), and transparency was measured with a Secchi disk. Additional water samples were stored at 4 °C and analyzed by Jiangxi Nanda Environmental Testing Co., Ltd. (Nanchang, China). Total nitrogen was measured by alkaline potassium persulfate digestion–ultraviolet spectrophotometry, total phosphorus by ammonium molybdate spectrophotometry, and ammonia nitrogen by Nessler’s reagent spectrophotometry. The permanganate index was determined by acidic titration with sodium oxalate, petroleum by infrared spectrophotometry, volatile phenol by 4-aminoantipyrine spectrophotometry, and copper by flameless atomic absorption spectrophotometry. Chlorophyll *a* was determined by ethanol extraction and spectrophotometry. Analytical methods, available detection limits, and environmental data are provided in Table S8.

Community counts were Hellinger-transformed. Environmental variables were standardized, and variance inflation factors (VIFs) above 10 were removed iteratively. Twelve candidate variables were screened; petroleum, copper, permanganate index, and ammonia nitrogen were removed in that order, leaving water temperature, pH, dissolved oxygen, total nitrogen, total phosphorus, volatile phenol, transparency, and chlorophyll *a* (Table S3). To limit model complexity with ten paired sites, standardized environmental variables were residualized against site and campaign and summarized by principal component analysis. The first two environmental components, selected without reference to fish-community significance, were used in partial RDA with site and campaign as conditioning variables. The overall test used reduced-model residual permutations restricted within site (1023 nonidentity exchanges). Eight separate single-variable partial RDAs used the same conditioning and permutation structure, with BH correction across the eight tests. The original eight-variable, campaign-conditioned model was retained only as a diagnostic (Table S3).

The ten taxa with the highest combined RRA were compared with the eight retained environmental variables across 20 site-by-campaign observations. For each taxon–variable pair, values were ranked and a partial-rank correlation was calculated after removing the campaign effect from both ranked variables. Approximate pointwise 95% confidence intervals were estimated from 3999 bootstrap resamples of the ten sites, keeping each site’s two campaign observations together. The correlations were used to describe taxon–environment patterns (Figure S1; Table S5).

### 2.8. Statistical Software

Analyses were performed in R 4.4.3 using vegan 2.6-10, ape 5.8-1, and permute 0.9-7. vegan functions included specnumber, diversity, rarefy, vegdist, adonis2, betadisper, permutest, rda, and anova.cca; PCoA used ape. Data handling and multiple-testing correction used base R (version 4.4.3) and stats (version 4.4.3), including p.adjust. ggplot2 (version 3.5.1) and ggrepel (version 0.9.6) were used for graphics and label placement.

## 3. Results

### 3.1. Sequencing and Technical-Replicate Quality Control

Two technical process replicates at site B1 had low fish-read counts: replicate 1 in 2023 had 13,351 reads (1.86% of the campaign median), and replicate 2 in 2024 had 527 reads (0.072%; Table S6). Both contributed only to pooled site profiles. The minimum pooled depth was 872,923 reads (Figure S2). Read-sum pooling and equal-weight averaging of replicate RRAs gave a median site-profile Bray–Curtis difference of 0.0075 (maximum 0.1127), and both retained the between-campaign compositional difference (Table S7). Filtering ≤5 reads within technical replicates reduced the combined inventory from 100 to 98 taxa, but the paired alpha-diversity tests remained nonsignificant and the campaign PERMANOVA tests remained significant. Presence–absence effect sizes were more sensitive to filtering than Bray–Curtis effect sizes.

### 3.2. Taxonomic Composition and Dominant Molecular Detection Signals

Ninety-six taxa were detected in 2023 and 94 in 2024. The combined inventory contained 100 fish taxa: 90 were detected in both surveys, six only in 2023, and four only in 2024. Six orders were detected during each survey; the 2023 inventory contained 16 families and 64 genera, and the 2024 inventory contained 15 families and 62 genera (Table 1; Table S1).

**Figure 2:**
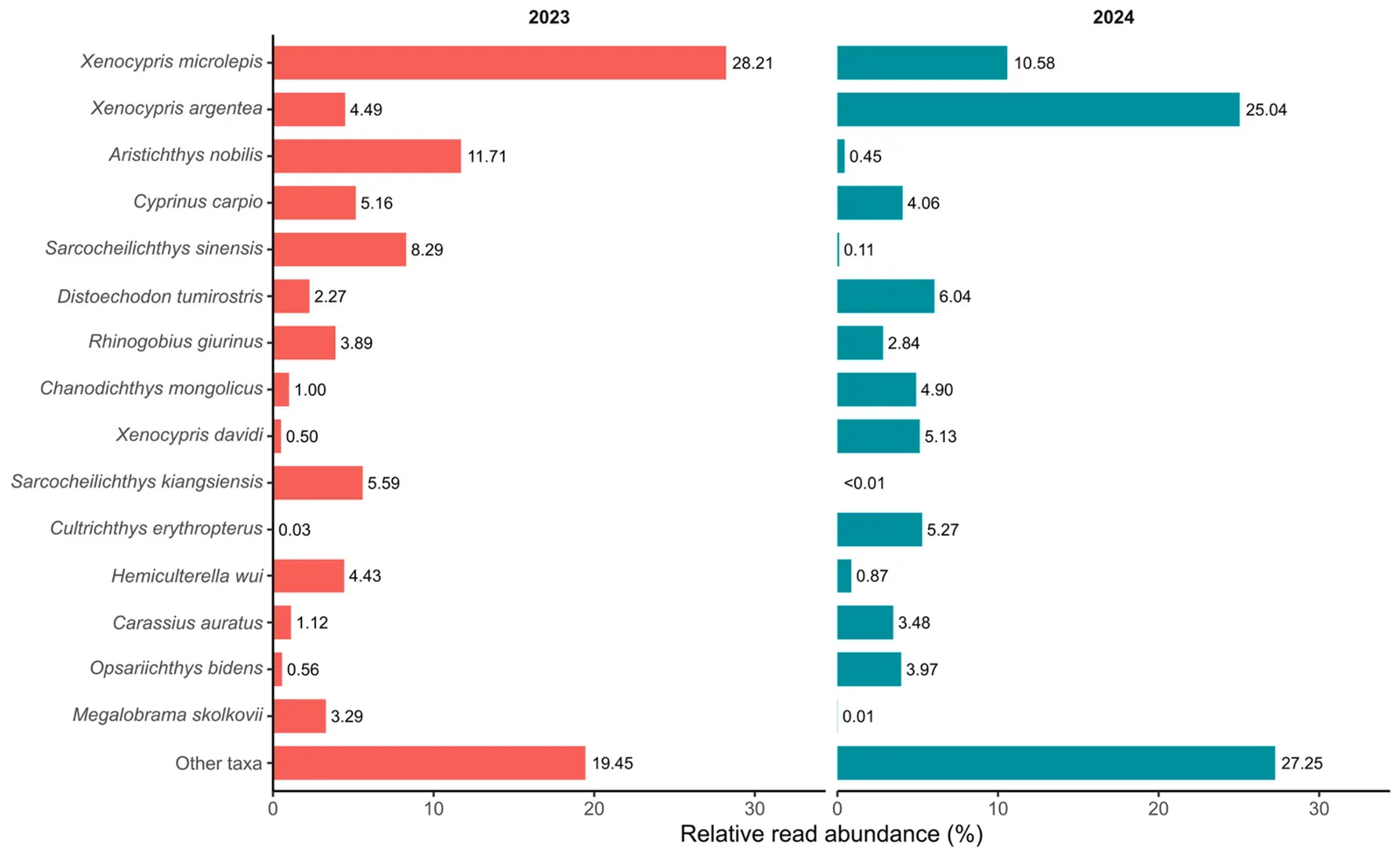
Relative read abundance (RRA) for the union of the ten highest-RRA fish taxa in each campaign. Both panels use the same taxon order. Other taxa pools all fish taxa outside this displayed union; labels show percentages. Read proportions are molecular detection signals, not estimates of fish abundance or biomass.

In 2023, *Xenocypris microlepis* produced the strongest molecular detection signal and accounted for 28.21% of fish reads. It was followed by *Aristichthys nobilis* (11.71%), *Sarcocheilichthys sinensis* (8.29%), *Sarcocheilichthys kiangsiensis* (5.59%), and *Cyprinus carpio* (5.16%). In 2024, *Xenocypris argentea* had the highest RRA (25.04%), followed by *X. microlepis* (10.58%), *Distoechodon tumirostris* (6.04%), *Cultrichthys erythropterus* (5.27%), and *Xenocypris davidi* (5.13%; Figure 2; Table S2). These proportions describe molecular detection signals, not abundance or biomass.

### 3.3. Alpha Diversity

**Figure 3:**
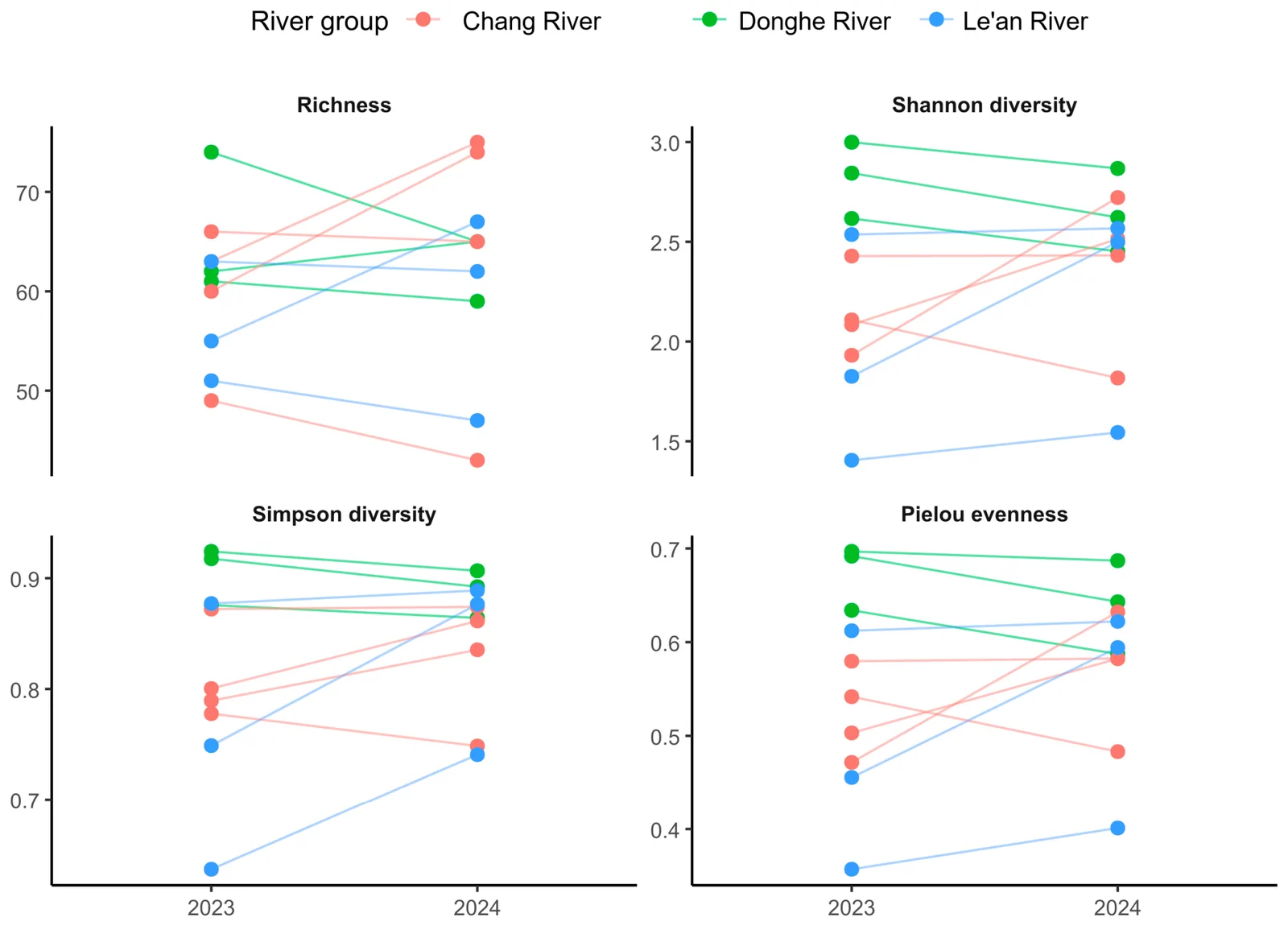
Site-level observed richness, Shannon diversity, Simpson diversity, and Pielou evenness in the two survey periods, grouped by river section. Lines connect the same sampling site across the two survey periods.

Mean site-level richness was 60.4 ± 7.3 taxa in 2023 and 62.2 ± 10.3 taxa in 2024. Mean rarefied richness was 55.85 ± 7.33 and 58.54 ± 9.32, respectively. Shannon diversity averaged 2.278 ± 0.494 in 2023 and 2.404 ± 0.408 in 2024; Simpson diversity averaged 0.822 ± 0.089 and 0.849 ± 0.058; and Pielou evenness averaged 0.554 ± 0.109 and 0.582 ± 0.083 (Table 1; Figure 3).

None of the five alpha-diversity measures differed between paired campaigns after BH correction (all adjusted *p* ≥ 0.420; paired *t*-tests, df = 9). Mean differences (2024 minus 2023) were 1.8 taxa for richness (95% CI −4.04 to 7.64), 2.69 taxa for rarefied richness (−3.29 to 8.67), 0.126 for Shannon diversity (−0.146 to 0.398), 0.0269 for Simpson diversity (−0.0129 to 0.0666), and 0.0271 for Pielou evenness (−0.0283 to 0.0826). No river-group alpha-diversity test was significant after correction (all adjusted *p* ≥ 0.251; Table S4).

### 3.4. Between-Campaign and River-Group Differences in Community Composition

**Figure 4:**
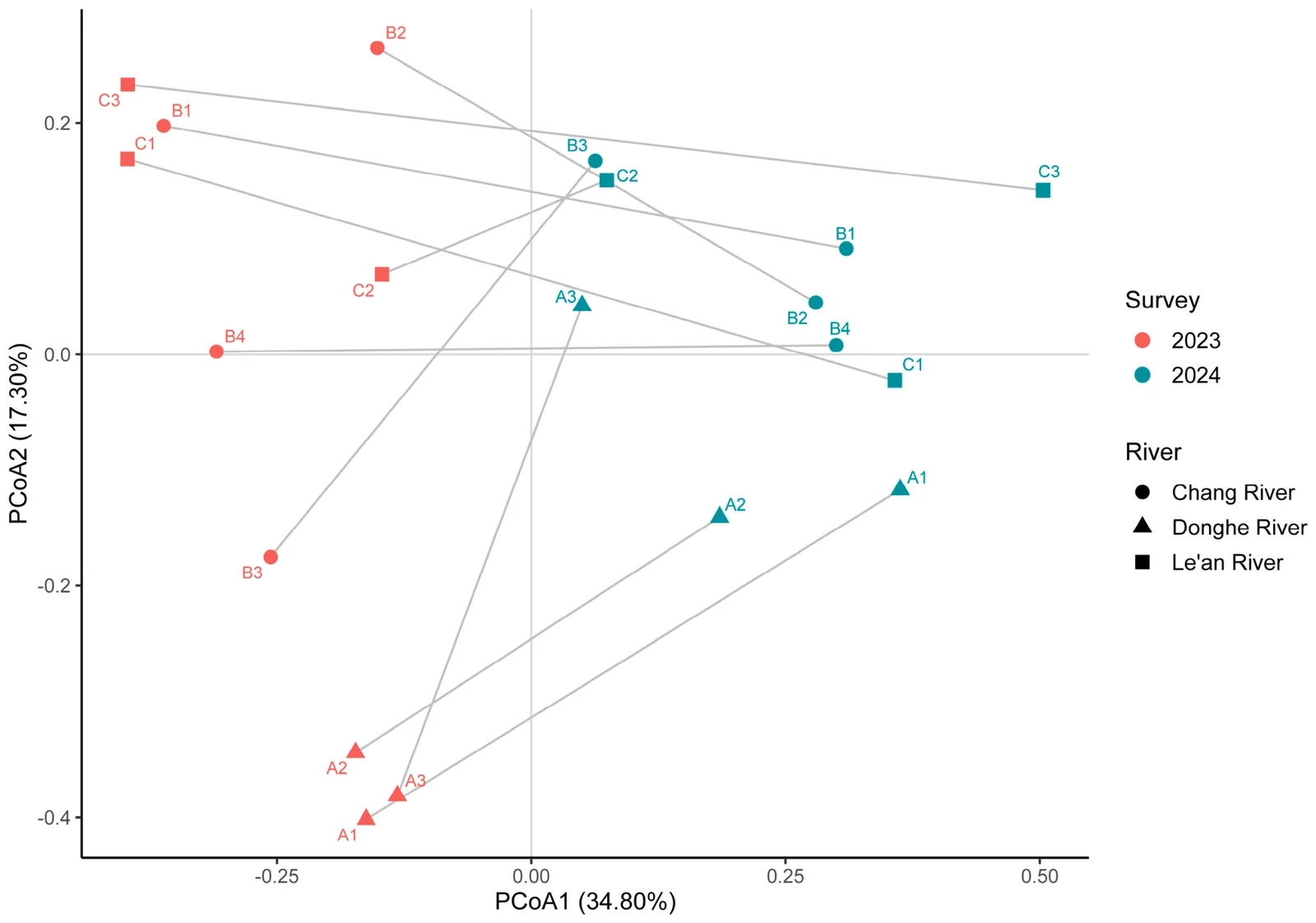
Principal coordinates analysis of site-level fish-community composition based on Bray–Curtis dissimilarity of RRA. The two axes explain 52.10% of variation. Colors denote campaign, symbols denote river, labels identify sites, and gray lines connect the same site across campaigns.

The first two Bray–Curtis PCoA axes explained 34.80% and 17.30% of variation, respectively (52.10% combined; Figure 4). Community composition differed between campaigns using Bray–Curtis (F_1,18_ = 7.466, R^2^ = 0.293, adjusted *p* = 0.002) and Jaccard dissimilarities (F_1,18_ = 4.095, R^2^ = 0.185, adjusted *p* = 0.002). Approximate paired-site bootstrap 95% intervals for R^2^ were 0.256–0.482 and 0.178–0.324, respectively. Campaign PERMDISP tests did not detect unequal dispersion (both adjusted *p* = 0.907), although their small sample size limits the ability to exclude dispersion differences.

River-group differentiation was clearer in 2023: Bray–Curtis F_2,7_ = 2.550, R^2^ = 0.421, adjusted *p* = 0.042; Jaccard F_2,7_ = 2.261, R^2^ = 0.392, adjusted *p* = 0.018. Neither 2024 comparison reached the 0.05 threshold after correction: Bray–Curtis F_2,7_ = 1.568, R^2^ = 0.309, adjusted *p* = 0.074; Jaccard F_2,7_ = 1.743, R^2^ = 0.333, adjusted *p* = 0.051. River-group PERMDISP tests were nonsignificant after correction (all adjusted *p* = 0.881; Table S4). These comparisons involved only three, four, and three sites per river group and should not be taken as evidence of sharply bounded assemblages.

### 3.5. Environmental Associations

**Figure 5:**
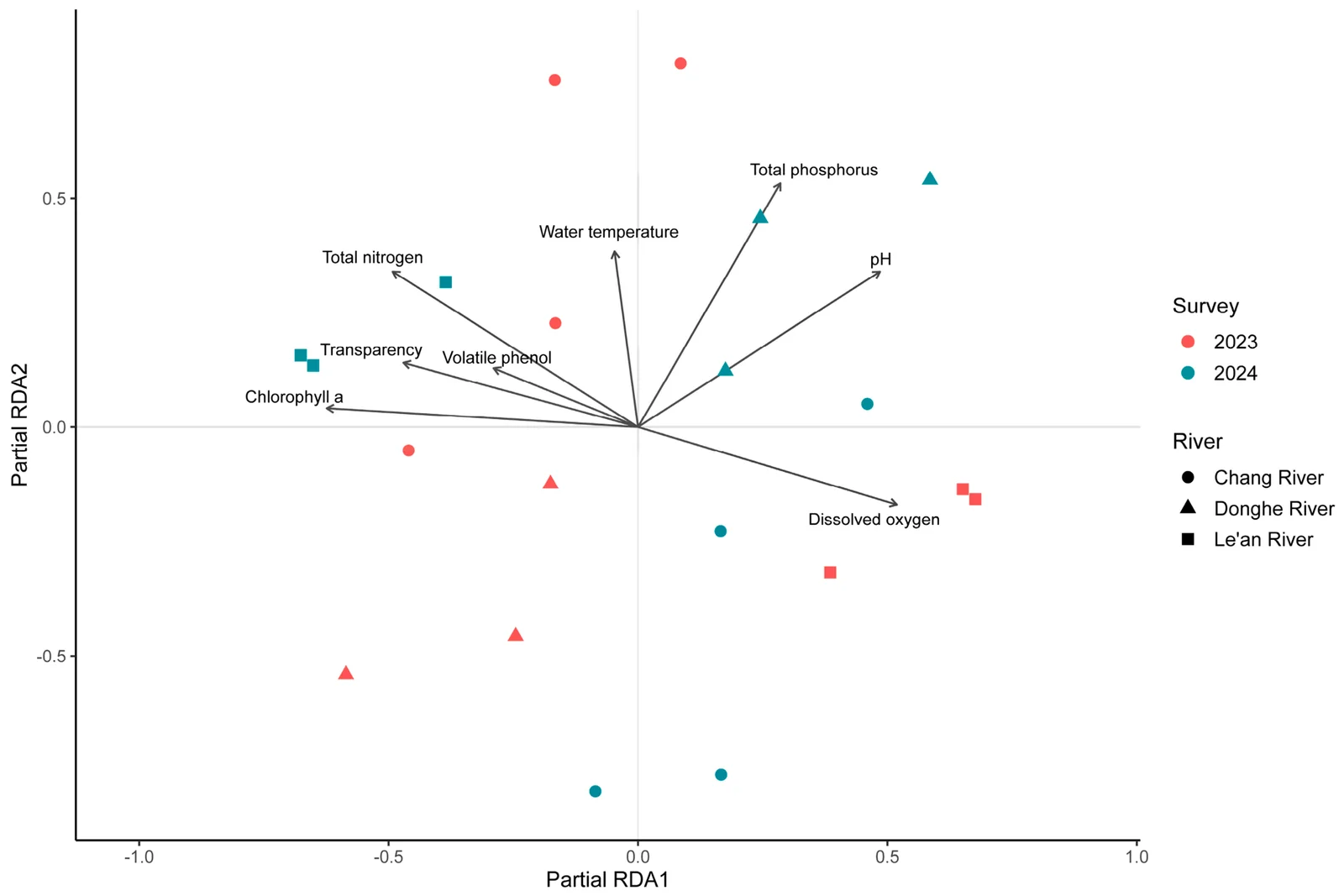
Partial RDA of Hellinger-transformed fish-community composition, constrained by two environmental principal components and conditioned on site and campaign (overall *p* = 0.420). Colors denote campaign and symbols denote river. Arrows show correlations of the eight residualized environmental variables with constrained ordination scores, rather than eight separately fitted predictors. Total nitrogen and total phosphorus are shown separately. Arrow directions are descriptive and do not establish independent effects.

After removing site and campaign effects, the first two environmental principal components accounted for 67.94% of residual environmental variation. The resulting partial RDA was not significant (F_2,7_ = 0.971, *p* = 0.420), with a constrained R^2^ of 0.0676 relative to total community variation and adjusted R^2^ of −0.0043 (Figure 5; Table S3). The negative adjusted value indicates no explanatory gain after the model-complexity adjustment, rather than negative biological variation.

None of the eight single-variable partial RDAs was significant after conditioning on site and campaign and correcting for multiple testing (all adjusted *p* ≥ 0.438; Table S3). The original eight-variable model conditioned only on campaign also remained nonsignificant with site-restricted permutations (F = 1.192, *p* = 0.394). These analyses provided no clear evidence of environmental associations independent of site and campaign effects.

The 80 campaign-adjusted taxon–environment partial-rank correlations ranged from −0.613 to 0.556 (Figure S1; Table S5). Table S5 reports the corresponding approximate site-cluster bootstrap confidence intervals.

## 4. Discussion

### 4.1. Fish Detections Across the Surveyed River Network

The two campaigns produced a combined inventory of 100 fish taxa, 90 of which were detected in both periods. This large shared component indicates recurrence at the level of the cross-site molecular inventory, but it should not be equated with complete stability of the underlying fish fauna. The ten period-specific taxa represented a small fraction of the inventory, and a single-period detection cannot by itself demonstrate colonization, disappearance, or a change in occupancy.

After harmonizing unambiguous names, 46 of 53 species in the middle Chang River survey, 35 of 38 at Fenghuang Bridge, and 34 of 40 in Wuxikou Reservoir matched our eDNA inventory [18,19,20]. This overlap supports regional plausibility. However, the studies differed in location, habitat, date, gear, and effort, and river-water eDNA may integrate DNA from upstream [14,15,27,28]. Unmatched names therefore do not demonstrate local absence, and the inventory totals cannot establish that either method detected more species under equivalent conditions.

Cypriniformes formed the largest component of each capture inventory (approximately 68–70% of recorded species) [18,19,20] and was likewise prominent in the eDNA dataset. This broad taxonomic agreement complements the species-level overlap without implying identical local assemblages.

### 4.2. Temporal Patterns in Local Diversity and Community Composition

Observed and rarefied richness, Shannon diversity, Simpson diversity, and Pielou evenness showed no significant paired differences between campaigns. In contrast, both Bray–Curtis and Jaccard analyses detected strong between-period differences, and PERMDISP did not detect unequal within-campaign dispersion. Alpha diversity summarizes the number and relative distribution of taxa within a site but does not retain their identities. Beta diversity is therefore able to detect replacement or redistribution of taxa and molecular signal even when local richness and evenness remain similar. The combined result supports a difference in community composition between the two survey datasets without a statistically detected study-wide difference in site-level diversity.

Comparable river studies have documented differences associated with flow, reproduction, migration, or habitat use [16,28,29]. Here, autumn 2023 and early summer 2024 represent two distinct survey campaigns. Season, year, and sampling-event effects cannot be separated by this design; the observed contrast is therefore interpreted as between-campaign compositional differentiation, not a general seasonal cycle.

### 4.3. Spatial Patterns Among River Groups Across Survey Periods

Spatial differentiation was evident but differed between campaigns. In 2023, both dissimilarity metrics distinguished river groups after BH correction; in 2024 neither comparison reached the corrected threshold. The Jaccard result in 2024 was close to that threshold and sensitive to the definition of the multiple-testing family, so it does not support a firm claim of river-group separation. Small river-group samples also limit inference about the persistence of spatial structure.

Riverine fish eDNA can preserve spatial structure while integrating signals beyond an individual sampling point [10,14]. Transport depends on discharge, channel form, degradation, and the distribution of DNA sources. Because discharge, velocity, and channel travel distances were not measured here, differences in spatial differentiation between campaigns cannot be attributed specifically to hydrological mixing or biological homogenization. The straight-line intersite distances describe sampling geometry, not measured eDNA transport distances.

### 4.4. Relative Detection Signals and Taxonomic Composition

The identity of the highest-RRA taxa changed markedly between campaigns. *X. microlepis* produced the strongest signal in 2023, whereas *X. argentea* did so in 2024. These changes help explain why Bray–Curtis composition differed even though 90 taxa were shared. Read proportions, however, combine the amount of template entering a sample with species-specific shedding, transport, degradation, primer affinity, PCR competition, and sequencing processes [30,31]. The high-RRA taxa should therefore be described as dominant molecular detection signals.

Metabarcoding can yield quantitative information when it is explicitly calibrated. In the Danube, combining metabarcoding proportions with total fish eDNA measured by qPCR and conventional abundance data produced useful large-scale estimates [32]. The present study lacked total-eDNA calibration, taxon-specific correction, and synchronous abundance or biomass measurements; its RRA values cannot be interpreted in the same way. This distinction is central to comparison with local capture surveys.

The Fenghuang Bridge survey identified *Carassius auratus*, *Acheilognathus imberbis*, *Sarcocheilichthys nigripinnis*, and *Squalidus argentatus* as dominant using an index of relative importance (IRI ≥ 1000) [19]. Wuxikou Reservoir reported high numerical proportions of *Hemiculter leucisculus* and *Sinibrama macrops*, whereas the species contributing strongly by weight were *Hypophthalmichthys molitrix*, *A. nobilis*, and *X. argentea* [20]. IRI combines occurrence, numerical proportion, and biomass proportion, while RRA is a compositional molecular measure. Agreement in the occurrence of common cyprinids supports broad community-level concordance; differences in rank are expected and should not be framed as contradictions.

### 4.5. Associations Between Community Patterns and Environmental Variables

The reduced environmental model and single-variable partial models did not detect associations independent of site and campaign effects. Accordingly, the data do not support identifying water temperature, dissolved oxygen, nutrients, transparency, or another measured variable as an independent driver of the fish eDNA assemblage.

Synchronous environmental sampling improved temporal alignment, but many environmental variables shifted together between campaigns. Accounting for repeated sites and reducing the number of predictors avoided fitting eight environmental terms to the limited residual information in twenty observations. The nonsignificant reduced model and near-zero adjusted R^2^ indicate limited evidence of independent environmental associations; they do not demonstrate the absence of environmental effects. The vectors in Figure 5 summarize covariation within this constrained space.

The taxon–environment correlations varied in direction and magnitude across taxa and measured variables (Figure S1; Table S5). They remain descriptive: the ten repeatedly sampled sites and two campaigns do not isolate the effects of individual variables, and RRA is a compositional molecular signal rather than a measure of fish abundance. Repeated same-season surveys would help separate environmental associations from other between-campaign changes.

River studies show that flow, temperature, pH, rainfall, longitudinal position, and biological season can influence eDNA-derived diversity in taxon- and system-specific ways [28,29]. Those findings justify measuring hydrology and habitat, but they do not make an unmeasured factor an explanation for the present result. Discharge, velocity, depth, channel width, substrate, riparian structure, floodplain connectivity, and reach-scale habitat complexity were absent from the routine dataset. Future work should combine repeated same-season sampling, direct flow measurements, habitat descriptors, and parallel capture surveys to separate local habitat associations from temporal changes in fish distribution, DNA production, and transport.

### 4.6. Study Scope, Limitations, and Implications for Monitoring

The study compared the same ten sites using a consistent sampling workflow in two campaigns and three connected river groups. Pooling technical process replicates avoided treating them as independent ecological observations, and site-restricted permutations addressed paired sampling. Rarefaction and low-read and equal-weight sensitivity analyses assessed selected aspects of robustness. This methodological consistency does not remove the difference in seasons and years between campaigns.

The inference is bounded by the study design. Ten sites do not represent the entire Raohe River basin, and two campaigns cannot separate seasonal, annual, and event-specific variation. Negative controls were screened by gel electrophoresis but not sequenced; absence of a visible band does not exclude low-level contamination. No positive control or mock community was included. Low-read sensitivity analyses support the main compositional contrast but cannot distinguish genuine rare detections from contamination. The short 12S marker and reference coverage may not resolve closely related fishes, and incompletely archived processing parameters and final sequence-to-taxon mappings limit full reconstruction of the original bioinformatic workflow. Riverine transport also limits localization of detections, and RRA was not calibrated against fish abundance or biomass.

The dataset provides a two-campaign molecular baseline for the ten surveyed sites, supporting future comparisons of detections, local diversity, and community composition. Such regional inventories contribute to the taxonomic and monitoring evidence base called for in multidisciplinary freshwater-conservation efforts [33,34], but this study did not test conservation outcomes [6,7]. Coordinated eDNA and standardized capture surveys would add voucher-based identification, size structure, abundance, and biomass. Repeated sampling in matched seasons, sequenced controls, improved regional reference sequences, and hydrological measurements would strengthen future ecological interpretation [10,11,28,29,32].

## 5. Conclusions

Environmental DNA metabarcoding detected 100 fish taxa across ten sites in two campaigns in the middle Raohe River system. Site-level alpha diversity did not differ significantly, whereas community composition differed between campaigns. These main patterns persisted under equal-weight technical-replicate averaging and low-read sensitivity analyses. River-group differences were clearer in 2023 and less conclusive in 2024 after multiple-testing correction. Environmental models accounting for repeated sites and campaign effects did not detect independent associations. The study establishes a two-campaign molecular baseline for the ten surveyed sites, not a seasonal time series or causal environmental assessment.

## Figures and Tables

**Table 1:** Fish-taxon inventory and site-level alpha diversity in the two campaigns. Values after ± are standard deviations among ten sites per campaign, not among technical replicates.

Metric	2023	2024
Fish taxa	96	94
Orders	6	6
Families	16	15
Genera	64	62
Site richness, mean ± SD	60.4 ± 7.3	62.2 ± 10.3
Rarefied richness, mean ± SD	55.85 ± 7.33	58.54 ± 9.32
Shannon diversity, mean ± SD	2.278 ± 0.494	2.404 ± 0.408
Simpson diversity, mean ± SD	0.822 ± 0.089	0.849 ± 0.058
Pielou evenness, mean ± SD	0.554 ± 0.109	0.582 ± 0.083

## Data Availability

The sequencing data generated in this study were deposited in the NCBI Sequence Read Archive under BioProject PRJNA1521089, BioSamples SAMN62788347–SAMN62788406, and SRA Run accessions SRR40454934–SRR40454993.

## Supplementary Materials

The following supporting information can be downloaded at: https://www.mdpi.com/article/10.3390/biology15201812/s1, Figure S1. Campaign-adjusted partial-rank correlations between the RRA of the ten taxa with the highest combined RRA and eight environmental variables across 20 site-by-campaign observations from ten repeatedly sampled sites; Figure S2. Site-level expected-richness rarefaction curves; Table S1. Fish taxa used in the analyses, with species and genus authorities and detections in each campaign; Table S2. Fish-taxon RRA and occurrence by campaign, with species and genus authorities; Table S3. RDA screening and constrained-model results; Table S4. Full alpha-diversity, beta-diversity (PERMANOVA), and PERMDISP outputs; Table S5. The ten taxa with the highest combined RRA and their 80 campaign-adjusted partial-rank correlations with eight environmental variables; Table S6. Read depth and richness of the 60 technical process replicates, with within-site, within-campaign technical-replicate Bray–Curtis dissimilarity in the continued table; Table S7. Low-read sensitivity analysis, with read-sum versus equal-weight RRA profiles in the continued table; Table S8. Site coordinates, with intersite distances, environmental measurement methods, available analytical detection limits, and environmental data in the continued tables.
