# Supplementary material for: Environmental DNA Metabarcoding Reveals Spatiotemporal Variation in Fish Communities of the Middle Raohe River, China
Source: Biology (Basel). 2026 Oct 10;15(20):1812. doi: 10.3390/biology15201812 (PMC13701769; doi:10.3390/biology15201812)
Supplement: Supplementary file 1 [file biology-15-01812-s001.zip › biology-4577314-supplementary.pdf]

Supplementary Materials

Figure S1. Campaign-adjusted partial-rank correlations between the RRA of the ten taxa with the highest combined RRA and eight environmental variables across 20 site-by-campaign observations from ten repeatedly sampled sites. Tile values and colors show correlation coefficients; no significance tests are displayed.

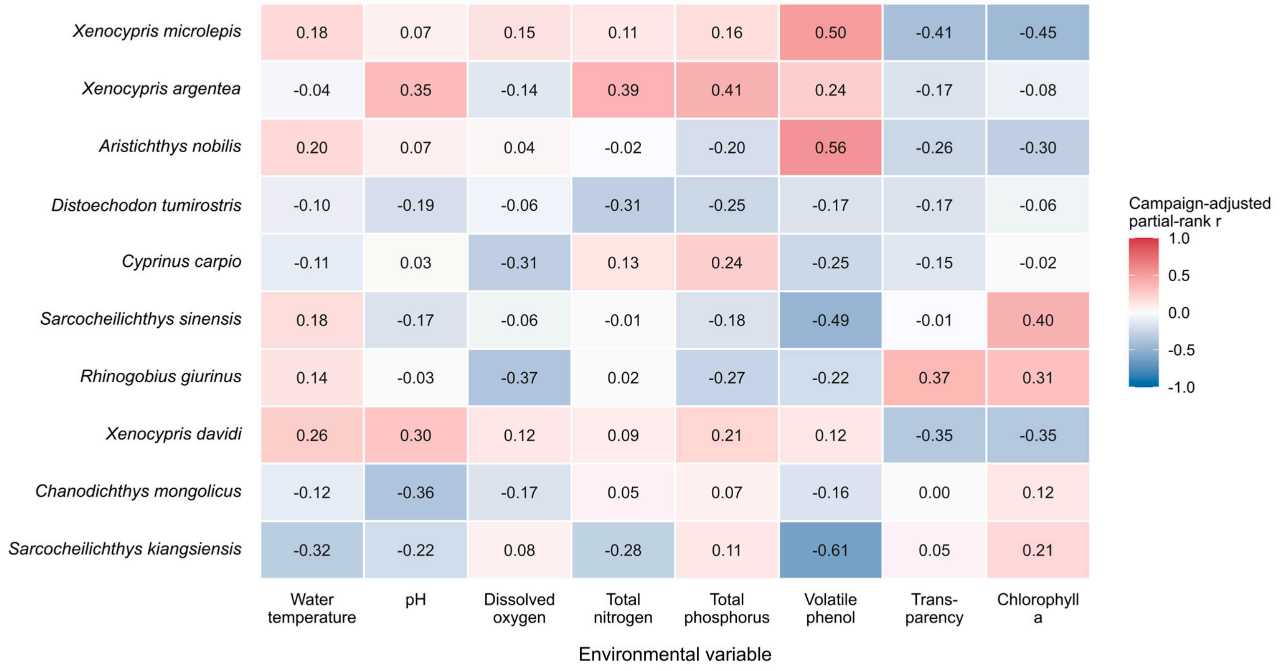

Figure S2. Site-level expected-richness rarefaction curves. The dashed line marks the common analytical rarefaction depth of 872,923 reads.

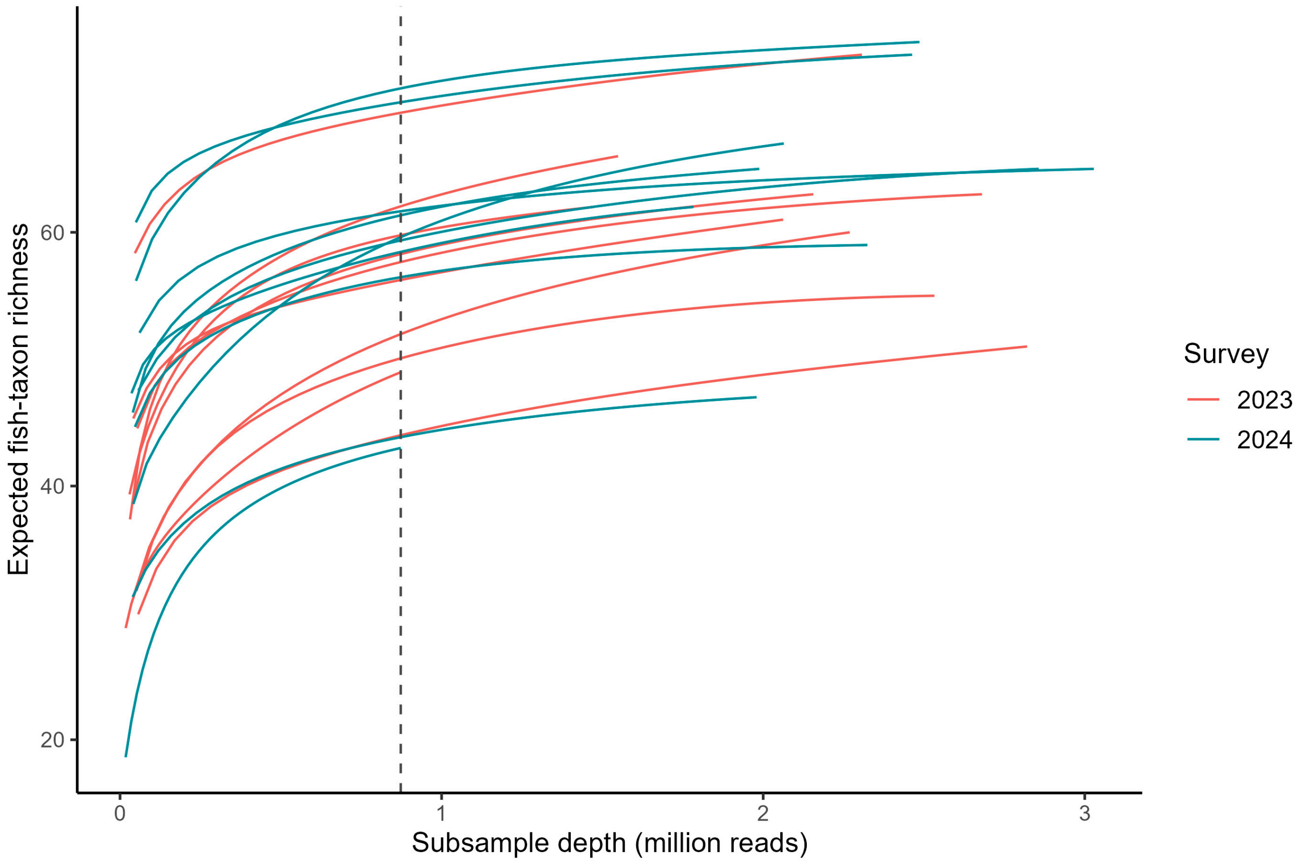

Table S1. Fish taxa used in the analyses, with species and genus authorities and detections in each campaign.

Parentheses around a species authority indicate a change from its original genus. Authorities are not italicized.

Taxon	Order	Family	Genus	Detected in 2023	Detected in 2024
Coilia brachygnathus Kreyenberg & Pappenheim, 1908	Clupeiformes	Engraulidae	*Coilia* Gray, 1830	Yes	Yes
Opsariichthys bidens Günther, 1873	Cypriniformes	Cyprinidae	*Opsariichthys* Bleeker, 1863	Yes	Yes
Zacco platypus (Temminck & Schlegel, 1846)	Cypriniformes	Cyprinidae	*Zacco* Jordan & Evermann, 1902	Yes	Yes
Rhynchocypris oxycephalus (Sauvage & Dabry, 1874)	Cypriniformes	Cyprinidae	*Rhynchocypris* Günther, 1889	Yes	Yes
Mylopharyngodon piceus (Richardson, 1846)	Cypriniformes	Cyprinidae	*Mylopharyngodon* Peters, 1881	Yes	Yes
Ctenopharyngodon Idella (Valenciennes, 1844)	Cypriniformes	Cyprinidae	*Ctenopharyngodon* Steindachner, 1866	Yes	Yes
Squaliobarbus curriculus (Richardson, 1846)	Cypriniformes	Cyprinidae	*Squaliobarbus* Günther, 1868	Yes	Yes
Ochetobius elongatus (Kner, 1867)	Cypriniformes	Cyprinidae	*Ochetobius* Günther, 1868	Yes	Yes
Elopichthys Bambusa (Richardson, 1845)	Cypriniformes	Cyprinidae	*Elopichthys* Bleeker, 1860	Yes	Yes
Sinibrama macrops (Günther, 1868)	Cypriniformes	Cyprinidae	*Sinibrama* Wu, 1939	Yes	Yes
Pseudolaubuca sinensis Bleeker, 1864	Cypriniformes	Cyprinidae	*Pseudolaubuca* Bleeker, 1865	Yes	Yes
Toxabramis swinhonis Günther, 1873	Cypriniformes	Cyprinidae	*Toxabramis* Günther, 1873	Yes	Yes
Hemiculter bleekeri Warpachowsky, 1887	Cypriniformes	Cyprinidae	*Hemiculter* Bleeker, 1860	Yes	Yes
Hemiculter leucisculus (Basilewsky, 1855)	Cypriniformes	Cyprinidae	*Hemiculter* Bleeker, 1860	Yes	Yes
Hemiculterella wui (Wang, 1935)	Cypriniformes	Cyprinidae	*Hemiculterella* Warpachowski, 1887	Yes	Yes
Pseudohemiculter dispar (Peters, 1880)	Cypriniformes	Cyprinidae	*Pseudohemiculter* Nichols & Pope, 1927	Yes	Yes
Culter Alburnus Basilewsky, 1855	Cypriniformes	Cyprinidae	*Culter* Basilewsky, 1855	Yes	Yes
Chanodichthys mongolicus (Basilewsky, 1855)	Cypriniformes	Cyprinidae	*Chanodichthys* Bleeker, 1860	Yes	Yes
Chanodichthys dabryi (Bleeker, 1871)	Cypriniformes	Cyprinidae	*Chanodichthys* Bleeker, 1860	Yes	Yes
Cultrichthys erythropterus (Basilewsky, 1855)	Cypriniformes	Cyprinidae	*Cultrichthys* Smith, 1938	Yes	Yes
Megalobrama skolkovii Dybowski, 1872	Cypriniformes	Cyprinidae	*Megalobrama* Dybowski, 1872	Yes	Yes
Megalobrama amblycephala Yih, 1955	Cypriniformes	Cyprinidae	*Megalobrama* Dybowski, 1872	Yes	Yes
Parabramis pekinensis (Basilewsky, 1855)	Cypriniformes	Cyprinidae	*Parabramis* Bleeker, 1864	Yes	Yes
Xenocypris argentea Günther, 1868	Cypriniformes	Cyprinidae	*Xenocypris* Günther, 1868	Yes	Yes

Taxon	Order	Family	Genus	Detected in 2023	Detected in 2024
Xenocypris davidi Bleeker, 1871	Cypriniformes	Cyprinidae	*Xenocypris* Günther, 1868	Yes	Yes
Xenocypris microlepis Bleeker, 1871	Cypriniformes	Cyprinidae	*Xenocypris* Günther, 1868	Yes	Yes
Distoechodon tumirostris Peters, 1880	Cypriniformes	Cyprinidae	*Distoechodon* Peters, 1880	Yes	Yes
Pseudobrama simoni (Bleeker, 1864)	Cypriniformes	Cyprinidae	*Pseudobrama* Bleeker, 1871	Yes	Yes
Hypophthalmichthys molitrix (Valenciennes, 1844)	Cypriniformes	Cyprinidae	*Hypophthalmichthys* Bleeker, 1860	Yes	Yes
Aristichthys nobilis (Richardson, 1845)	Cypriniformes	Cyprinidae	*Aristichthys* Oshima, 1919	Yes	Yes
Rhodeus ocellatus (Kner, 1867)	Cypriniformes	Cyprinidae	*Rhodeus* Agassiz, 1835	Yes	Yes
Rhodeus sinensis Günther, 1868	Cypriniformes	Cyprinidae	*Rhodeus* Agassiz, 1835	Yes	Yes
Acheilognathus barbatulus Günther, 1873	Cypriniformes	Cyprinidae	*Acheilognathus* Bleeker, 1860	Yes	Yes
Acheilognathus barbatus Nichols, 1926	Cypriniformes	Cyprinidae	*Acheilognathus* Bleeker, 1860	Yes	Yes
Acheilognathus chankaensis (Dybowski, 1872)	Cypriniformes	Cyprinidae	*Acheilognathus* Bleeker, 1860	No	Yes
Acheilognathus hypselonotus (Bleeker, 1871)	Cypriniformes	Cyprinidae	*Acheilognathus* Bleeker, 1860	Yes	Yes
Acheilognathus imberbis Günther, 1868	Cypriniformes	Cyprinidae	*Acheilognathus* Bleeker, 1860	Yes	Yes
Acheilognathus macropterus (Bleeker, 1871)	Cypriniformes	Cyprinidae	*Acheilognathus* Bleeker, 1860	Yes	Yes
Acheilognathus rhombeus (Temminck & Schlegel, 1846)	Cypriniformes	Cyprinidae	*Acheilognathus* Bleeker, 1860	Yes	Yes
Acheilognathus tonkinensis (Vaillant, 1892)	Cypriniformes	Cyprinidae	*Acheilognathus* Bleeker, 1860	Yes	Yes
Hemibarbus labeo (Pallas, 1776)	Cypriniformes	Cyprinidae	*Hemibarbus* Bleeker, 1860	Yes	Yes
Hemibarbus maculatus Bleeker, 1871	Cypriniformes	Cyprinidae	*Hemibarbus* Bleeker, 1860	Yes	Yes
Pseudorasbora elongata Wu, 1939	Cypriniformes	Cyprinidae	*Pseudorasbora* Bleeker, 1860	Yes	Yes
Pseudorasbora parva (Temminck & Schlegel, 1846)	Cypriniformes	Cyprinidae	*Pseudorasbora* Bleeker, 1860	Yes	Yes
Sarcocheilichthys nigripinnis (Günther, 1873)	Cypriniformes	Cyprinidae	*Sarcocheilichthys* Bleeker, 1860	Yes	Yes
Sarcocheilichthys parvus Nichols, 1930	Cypriniformes	Cyprinidae	*Sarcocheilichthys* Bleeker, 1860	Yes	Yes
Sarcocheilichthys kiangsiensis Nichols, 1930	Cypriniformes	Cyprinidae	*Sarcocheilichthys* Bleeker, 1860	Yes	Yes
Sarcocheilichthys sinensis Bleeker, 1871	Cypriniformes	Cyprinidae	*Sarcocheilichthys* Bleeker, 1860	Yes	Yes
Gnathopogon imberbis (Sauvage & Dabry, 1874)	Cypriniformes	Cyprinidae	*Gnathopogon* Bleeker, 1860	Yes	Yes
Squalidus argentatus (Sauvage & Dabry, 1874)	Cypriniformes	Cyprinidae	*Squalidus* Dybowski, 1872	Yes	Yes

**Taxon**	**Order**	**Family**	**Genus**	**Detected in 2023**	**Detected in 2024**
Abbottina rivularis (Basilewsky, 1855)	Cypriniformes	Cyprinidae	*Abbottina* Jordan & Fowler, 1903	Yes	Yes
Microphysogobio fukiensis (Nichols, 1926)	Cypriniformes	Cyprinidae	*Microphysogobio* Mori, 1933	Yes	Yes
Pseudogobio vaillanti (Sauvage, 1878)	Cypriniformes	Cyprinidae	*Pseudogobio* Bleeker, 1860	Yes	Yes
Saurogobio dabryi Bleeker, 1871	Cypriniformes	Cyprinidae	*Saurogobio* Bleeker, 1870	Yes	Yes
Saurogobio dumerili Bleeker, 1871	Cypriniformes	Cyprinidae	*Saurogobio* Bleeker, 1870	Yes	Yes
Gobiobotia filifer (Garman, 1912)	Cypriniformes	Cyprinidae	*Gobiobotia* Kreyenberg, 1911	Yes	Yes
Gobiobotia meridionalis Chen & Tsao, 1982	Cypriniformes	Cyprinidae	*Gobiobotia* Kreyenberg, 1911	No	Yes
Spinibarbus hollandi Oshima, 1919	Cypriniformes	Cyprinidae	*Spinibarbus* Oshima, 1919	Yes	Yes
Acrossocheilus kreyenbergii (Regan, 1908)	Cypriniformes	Cyprinidae	*Acrossocheilus* Oshima, 1919	Yes	Yes
Cirrhinus molitorella (Valenciennes, 1844)	Cypriniformes	Cyprinidae	*Cirrhinus* Oken, 1817	Yes	Yes
Cirrhinus mrigala (Hamilton, 1822)	Cypriniformes	Cyprinidae	*Cirrhinus* Oken, 1817	Yes	No
Cyprinus carpio Linnaeus, 1758	Cypriniformes	Cyprinidae	*Cyprinus* Linnaeus, 1758	Yes	Yes
Carassius auratus (Linnaeus, 1758)	Cypriniformes	Cyprinidae	*Carassius* Jarocki, 1822	Yes	Yes
Parabotia banarescui (Nalbant, 1965)	Cypriniformes	Cobitidae	*Parabotia* Dabry de Thiersant, 1872	Yes	Yes
Parabotia fasciata Dabry de Thiersant, 1872	Cypriniformes	Cobitidae	*Parabotia* Dabry de Thiersant, 1872	Yes	Yes
Parabotia kiangsiensis Liu & Guo, 1986	Cypriniformes	Cobitidae	*Parabotia* Dabry de Thiersant, 1872	Yes	Yes
Parabotia lijiangensis Chen, 1980	Cypriniformes	Cobitidae	*Parabotia* Dabry de Thiersant, 1872	Yes	Yes
Misgurnus anguillicaudatus (Cantor, 1842)	Cypriniformes	Cobitidae	*Misgurnus* Lacépède, 1803	Yes	Yes
Paramisgurnus dabryanus Dabry de Thiersant, 1872	Cypriniformes	Cobitidae	*Paramisgurnus* Dabry de Thiersant, 1872	Yes	Yes
Leptobotia tientainensis (Wu, 1930)	Cypriniformes	Cobitidae	*Leptobotia* Bleeker, 1870	No	Yes
Cobitis sinensis Sauvage & Dabry de Thiersant, 1874	Cypriniformes	Cobitidae	*Cobitis* Linnaeus, 1758	Yes	Yes
Vanmanenia pingchowensis (Fang, 1935)	Cypriniformes	Balitoridae	*Vanmanenia* Hora, 1932	Yes	Yes
Pelteobagrus eupogon (Boulenger, 1892)	Siluriformes	Bagridae	*Pelteobagrus* Bleeker, 1864	Yes	Yes
Pelteobagrus fulvidraco (Richardson, 1846)	Siluriformes	Bagridae	*Pelteobagrus* Bleeker, 1864	Yes	Yes
Pelteobagrus nitidus (Sauvage & Dabry, 1874)	Siluriformes	Bagridae	*Pelteobagrus* Bleeker, 1864	Yes	Yes
Pelteobagrus vachellii (Richardson, 1846)	Siluriformes	Bagridae	*Pelteobagrus* Bleeker, 1864	No	Yes

**Taxon**	**Order**	**Family**	**Genus**	**Detected in 2023**	**Detected in 2024**
Leiocassis crassilabris Günther, 1864	Siluriformes	Bagridae	*Leiocassis* Bleeker, 1857	Yes	Yes
Pseudobagrus tenuis (Günther, 1873)	Siluriformes	Bagridae	*Pseudobagrus* Bleeker, 1858	Yes	No
Pseudobagrus truncatus (Regan, 1913)	Siluriformes	Bagridae	*Pseudobagrus* Bleeker, 1858	Yes	Yes
Hemibagrus macropterus (Bleeker, 1870)	Siluriformes	Bagridae	*Hemibagrus* Bleeker, 1862	Yes	No
Liobagrus styani Regan, 1908	Siluriformes	Amblycipitidae	*Liobagrus* Hilgendorf, 1878	Yes	Yes
Silurus asotus Linnaeus, 1758	Siluriformes	Siluridae	*Silurus* Linnaeus, 1758	Yes	Yes
Neosalanx taihuensis Chen, 1956	Osmeriformes	Salangidae	*Neosalanx* Wakiya & Takahasi, 1937	Yes	Yes
Protosalanx chinensis (Basilewsky, 1855)	Osmeriformes	Salangidae	*Protosalanx* Regan, 1908	Yes	No
Siniperca chuatsi (Basilewsky, 1855)	Perciformes	Percichthyidae	*Siniperca* Gill, 1862	Yes	Yes
Siniperca knerii Garman, 1912	Perciformes	Percichthyidae	*Siniperca* Gill, 1862	Yes	Yes
Siniperca roulei Wu, 1930	Perciformes	Percichthyidae	*Siniperca* Gill, 1862	Yes	Yes
Siniperca scherzeri Steindachner, 1892	Perciformes	Percichthyidae	*Siniperca* Gill, 1862	Yes	Yes
Siniperca undulata Fang & Chong, 1932	Perciformes	Percichthyidae	*Siniperca* Gill, 1862	Yes	Yes
Odontobutis sinensis Wu, Chen & Chong, 2002	Perciformes	Odontobutidae	*Odontobutis* Bleeker, 1874	Yes	Yes
Odontobutis potamophila (Günther, 1861)	Perciformes	Odontobutidae	*Odontobutis* Bleeker, 1874	Yes	No
Micropercops swinhonis (Günther, 1873)	Perciformes	Odontobutidae	*Micropercops* Fowler & Bean, 1920	Yes	Yes
Rhinogobius brunneus (Temminck & Schlegel, 1845)	Perciformes	Gobiidae	*Rhinogobius* Gill, 1859	Yes	Yes
Rhinogobius cliffordpopei (Nichols, 1925)	Perciformes	Gobiidae	*Rhinogobius* Gill, 1859	Yes	Yes
Rhinogobius giurinus (Rutter, 1897)	Perciformes	Gobiidae	*Rhinogobius* Gill, 1859	Yes	Yes
Macropodus opercularis (Linnaeus, 1758)	Perciformes	Osphronemidae	*Macropodus* Lacépède, 1801	Yes	No
Channa argus (Cantor, 1842)	Perciformes	Channidae	*Channa* Scopoli, 1777	Yes	Yes
Micropterus salmoides (Lacépède, 1802)	Perciformes	Centrarchidae	*Micropterus* Lacépède, 1802	Yes	Yes
Coptodon zillii (Gervais, 1848)	Perciformes	Cichlidae	*Coptodon* Gervais, 1853	Yes	Yes
Sinobdella sinensis (Bleeker, 1870)	Synbranchiformes	Mastacembelidae	*Sinobdella* Kottelat & Lim, 1994	Yes	Yes

Table S2. Fish-taxon RRA and occurrence by campaign, with species and genus authorities.

Table S2. 2023 campaign.

Year	Rank	Taxon and authority	Order	Family	Genus and authority	Reads	RRA	Sites	Frequency
2023	73	*Coilia brachygnathus* Kreyenberg & Pappenheim, 1908	Clupeiformes	Engraulidae	*Coilia* Gray, 1830	1562	7.54e-05	10	1.00
2023	24	*Opsariichthys bidens* Günther, 1873	Cypriniformes	Cyprinidae	*Opsariichthys* Bleeker, 1863	116850	0.00564	9	0.90
2023	21	*Zacco platypus* (Temminck & Schlegel, 1846)	Cypriniformes	Cyprinidae	*Zacco* Jordan & Evermann, 1902	164132	0.00792	9	0.90
2023	58	*Rhynchocypris oxycephalus* (Sauvage & Dabry, 1874)	Cypriniformes	Cyprinidae	*Rhynchocypris* Günther, 1889	9694	0.000468	1	0.10
2023	67	*Mylopharyngodon piceus* (Richardson, 1846)	Cypriniformes	Cyprinidae	*Mylopharyngodon* Peters, 1881	5021	0.000242	9	0.90
2023	20	*Ctenopharyngodon Idella* (Valenciennes, 1844)	Cypriniformes	Cyprinidae	*Ctenopharyngodon* Steindachner, 1866	180663	0.00872	10	1.00
2023	45	*Squaliobarbus curriculus* (Richardson, 1846)	Cypriniformes	Cyprinidae	*Squaliobarbus* Günther, 1868	23381	0.00113	10	1.00
2023	82	*Ochetobius elongatus* (Kner, 1867)	Cypriniformes	Cyprinidae	*Ochetobius* Günther, 1868	109	5.26e-06	4	0.40
2023	29	*Elopichthys Bambusa* (Richardson, 1845)	Cypriniformes	Cyprinidae	*Elopichthys* Bleeker, 1860	88345	0.00426	8	0.80
2023	88	*Sinibrama macrops* (Günther, 1868)	Cypriniformes	Cyprinidae	*Sinibrama* Wu, 1939	30	1.45e-06	1	0.10
2023	32	*Pseudolaubuca sinensis* Bleeker, 1864	Cypriniformes	Cyprinidae	*Pseudolaubuca* Bleeker, 1865	70614	0.00341	7	0.70
2023	76	*Toxabramis swinhonis* Günther, 1873	Cypriniformes	Cyprinidae	*Toxabramis* Günther, 1873	641	3.09e-05	7	0.70
2023	35	*Hemiculter bleekeri* Warpachowsky, 1887	Cypriniformes	Cyprinidae	*Hemiculter* Bleeker, 1860	63122	0.00305	10	1.00
2023	22	*Hemiculter leucisculus* (Basilewsky, 1855)	Cypriniformes	Cyprinidae	*Hemiculter* Bleeker, 1860	149620	0.00722	10	1.00
2023	7	*Hemiculterella wui* (Wang, 1935)	Cypriniformes	Cyprinidae	*Hemiculterella* Warpachowski, 1887	918417	0.0443	10	1.00
2023	49	*Pseudohemiculter dispar* (Peters, 1880)	Cypriniformes	Cyprinidae	*Pseudohemiculter* Nichols & Pope, 1927	17160	0.000828	9	0.90
2023	42	*Culter Alburnus* Basilewsky, 1855	Cypriniformes	Cyprinidae	*Culter* Basilewsky, 1855	36207	0.00175	10	1.00
2023	19	*Chanodichthys mongolicus* (Basilewsky, 1855)	Cypriniformes	Cyprinidae	*Chanodichthys* Bleeker, 1860	207113	0.00999	10	1.00

Year	Rank	Taxon and authority	Order	Family	Genus and authority	Reads	RRA	Sites	Frequency
2023	77	*Chanodichthys dabryi* (Bleeker, 1871)	Cypriniformes	Cyprinidae	*Chanodichthys* Bleeker, 1860	590	2.85e-05	8	0.80
2023	63	*Cultrichthys erythropterus* (Basilewsky, 1855)	Cypriniformes	Cyprinidae	*Cultrichthys* Smith, 1938	7174	0.000346	10	1.00
2023	9	*Megalobrama skolkovii* Dybowski, 1872	Cypriniformes	Cyprinidae	*Megalobrama* Dybowski, 1872	682831	0.0329	7	0.70
2023	66	*Megalobrama amblycephala* Yih, 1955	Cypriniformes	Cyprinidae	*Megalobrama* Dybowski, 1872	5522	0.000266	9	0.90
2023	36	*Parabramis pekinensis* (Basilewsky, 1855)	Cypriniformes	Cyprinidae	*Parabramis* Bleeker, 1864	61160	0.00295	10	1.00
2023	6	*Xenocypris argentea* Günther, 1868	Cypriniformes	Cyprinidae	*Xenocypris* Günther, 1868	931358	0.0449	10	1.00
2023	27	*Xenocypris davidi* Bleeker, 1871	Cypriniformes	Cyprinidae	*Xenocypris* Günther, 1868	104213	0.00503	10	1.00
2023	1	*Xenocypris microlepis* Bleeker, 1871	Cypriniformes	Cyprinidae	*Xenocypris* Günther, 1868	5846487	0.282	10	1.00
2023	10	*Distoechodon tumirostris* Peters, 1880	Cypriniformes	Cyprinidae	*Distoechodon* Peters, 1880	470026	0.0227	10	1.00
2023	83	*Pseudobrama simoni* (Bleeker, 1864)	Cypriniformes	Cyprinidae	*Pseudobrama* Bleeker, 1871	78	3.76e-06	6	0.60
2023	28	*Hypophthalmichthys molitrix* (Valenciennes, 1844)	Cypriniformes	Cyprinidae	*Hypophthalmichthys* Bleeker, 1860	88775	0.00428	10	1.00
2023	2	*Aristichthys nobilis* (Richardson, 1845)	Cypriniformes	Cyprinidae	*Aristichthys* Oshima, 1919	2425919	0.117	10	1.00
2023	14	*Rhodeus ocellatus* (Kner, 1867)	Cypriniformes	Cyprinidae	*Rhodeus* Agassiz, 1835	256552	0.0124	7	0.70
2023	48	*Rhodeus sinensis* Günther, 1868	Cypriniformes	Cyprinidae	*Rhodeus* Agassiz, 1835	17558	0.000847	4	0.40
2023	85	*Acheilognathus barbatulus* Günther, 1873	Cypriniformes	Cyprinidae	*Acheilognathus* Bleeker, 1860	52	2.51e-06	3	0.30
2023	41	*Acheilognathus barbatus* Nichols, 1926	Cypriniformes	Cyprinidae	*Acheilognathus* Bleeker, 1860	36527	0.00176	8	0.80
2023	97	*Acheilognathus chankaensis* (Dybowski, 1872)	Cypriniformes	Cyprinidae	*Acheilognathus* Bleeker, 1860	0	0	0	0.00
2023	86	*Acheilognathus hypselonotus* (Bleeker, 1871)	Cypriniformes	Cyprinidae	*Acheilognathus* Bleeker, 1860	49	2.36e-06	4	0.40
2023	47	*Acheilognathus imberbis* Günther, 1868	Cypriniformes	Cyprinidae	*Acheilognathus* Bleeker, 1860	17938	0.000866	4	0.40
2023	79	*Acheilognathus macropterus* (Bleeker, 1871)	Cypriniformes	Cyprinidae	*Acheilognathus* Bleeker, 1860	282	1.36e-05	6	0.60

Year	Rank	Taxon and authority	Order	Family	Genus and authority	Reads	RRA	Sites	Frequency
2023	11	*Acheilognathus rhombeus* (Temminck & Schlegel, 1846)	Cypriniformes	Cyprinidae	*Acheilognathus* Bleeker, 1860	395586	0.0191	10	1.00
2023	91	*Acheilognathus tonkinensis* (Vaillant, 1892)	Cypriniformes	Cyprinidae	*Acheilognathus* Bleeker, 1860	9	4.34e-07	1	0.10
2023	71	*Hemibarbus labeo* (Pallas, 1776)	Cypriniformes	Cyprinidae	*Hemibarbus* Bleeker, 1860	2642	0.000127	9	0.90
2023	12	*Hemibarbus maculatus* Bleeker, 1871	Cypriniformes	Cyprinidae	*Hemibarbus* Bleeker, 1860	294162	0.0142	10	1.00
2023	54	*Pseudorasbora elongata* Wu, 1939	Cypriniformes	Cyprinidae	*Pseudorasbora* Bleeker, 1860	13622	0.000657	4	0.40
2023	38	*Pseudorasbora parva* (Temminck & Schlegel, 1846)	Cypriniformes	Cyprinidae	*Pseudorasbora* Bleeker, 1860	43630	0.00211	9	0.90
2023	43	*Sarcocheilichthys nigripinnis* (Günther, 1873)	Cypriniformes	Cyprinidae	*Sarcocheilichthys* Bleeker, 1860	28010	0.00135	6	0.60
2023	64	*Sarcocheilichthys parvus* Nichols, 1930	Cypriniformes	Cyprinidae	*Sarcocheilichthys* Bleeker, 1860	6993	0.000337	5	0.50
2023	4	*Sarcocheilichthys kiangsiensis* Nichols, 1930	Cypriniformes	Cyprinidae	*Sarcocheilichthys* Bleeker, 1860	1157770	0.0559	10	1.00
2023	3	*Sarcocheilichthys sinensis* Bleeker, 1871	Cypriniformes	Cyprinidae	*Sarcocheilichthys* Bleeker, 1860	1718080	0.0829	10	1.00
2023	89	*Gnathopogon imberbis* (Sauvage & Dabry, 1874)	Cypriniformes	Cyprinidae	*Gnathopogon* Bleeker, 1860	26	1.25e-06	4	0.40
2023	13	*Squalidus argentatus* (Sauvage & Dabry, 1874)	Cypriniformes	Cyprinidae	*Squalidus* Dybowski, 1872	293363	0.0142	10	1.00
2023	40	*Abbottina rivularis* (Basilewsky, 1855)	Cypriniformes	Cyprinidae	*Abbottina* Jordan & Fowler, 1903	38070	0.00184	10	1.00
2023	25	*Microphysogobio fukiensis* (Nichols, 1926)	Cypriniformes	Cyprinidae	*Microphysogobio* Mori, 1933	110493	0.00533	10	1.00
2023	53	*Pseudogobio vaillanti* (Sauvage, 1878)	Cypriniformes	Cyprinidae	*Pseudogobio* Bleeker, 1860	14529	0.000701	5	0.50
2023	30	*Saurogobio dabryi* Bleeker, 1871	Cypriniformes	Cyprinidae	*Saurogobio* Bleeker, 1870	80333	0.00388	8	0.80
2023	96	*Saurogobio dumerili* Bleeker, 1871	Cypriniformes	Cyprinidae	*Saurogobio* Bleeker, 1870	2	9.65e-08	1	0.10
2023	80	*Gobiobotia filifer* (Garman, 1912)	Cypriniformes	Cyprinidae	*Gobiobotia* Kreyenberg, 1911	195	9.41e-06	8	0.80
2023	98	*Gobiobotia meridionalis* Chen & Tsao, 1982	Cypriniformes	Cyprinidae	*Gobiobotia* Kreyenberg, 1911	0	0	0	0.00
2023	23	*Spinibarbus hollandi* Oshima, 1919	Cypriniformes	Cyprinidae	*Spinibarbus* Oshima, 1919	119038	0.00574	9	0.90

Year	Rank	Taxon and authority	Order	Family	Genus and authority	Reads	RRA	Sites	Frequency
2023	17	*Acrossocheilus kreyenbergii* (Regan, 1908)	Cypriniformes	Cyprinidae	*Acrossocheilus* Oshima, 1919	230930	0.0111	8	0.80
2023	60	*Cirrhinus molitorella* (Valenciennes, 1844)	Cypriniformes	Cyprinidae	*Cirrhinus* Oken, 1817	8100	0.000391	2	0.20
2023	87	*Cirrhinus mrigala* (Hamilton, 1822)	Cypriniformes	Cyprinidae	*Cirrhinus* Oken, 1817	36	1.74e-06	2	0.20
2023	5	*Cyprinus carpio* Linnaeus, 1758	Cypriniformes	Cyprinidae	*Cyprinus* Linnaeus, 1758	1068481	0.0516	10	1.00
2023	16	*Carassius auratus* (Linnaeus, 1758)	Cypriniformes	Cyprinidae	*Carassius* Jarocki, 1822	232133	0.0112	10	1.00
2023	75	*Parabotia banarescui* (Nalbant, 1965)	Cypriniformes	Cobitidae	*Parabotia* Dabry de Thiersant, 1872	1023	4.94e-05	1	0.10
2023	33	*Parabotia fasciata* Dabry de Thiersant, 1872	Cypriniformes	Cobitidae	*Parabotia* Dabry de Thiersant, 1872	69359	0.00335	4	0.40
2023	81	*Parabotia kiangsiensis* Liu & Guo, 1986	Cypriniformes	Cobitidae	*Parabotia* Dabry de Thiersant, 1872	184	8.88e-06	1	0.10
2023	74	*Parabotia lijiangensis* Chen, 1980	Cypriniformes	Cobitidae	*Parabotia* Dabry de Thiersant, 1872	1196	5.77e-05	4	0.40
2023	69	*Misgurnus anguillicaudatus* (Cantor, 1842)	Cypriniformes	Cobitidae	*Misgurnus* Lacépède, 1803	3260	0.000157	2	0.20
2023	52	*Paramisgurnus dabryanus* Dabry de Thiersant, 1872	Cypriniformes	Cobitidae	*Paramisgurnus* Dabry de Thiersant, 1872	15905	0.000767	5	0.50
2023	99	*Leptobotia tientainensis* (Wu, 1930)	Cypriniformes	Cobitidae	*Leptobotia* Bleeker, 1870	0	0	0	0.00
2023	57	*Cobitis sinensis* Sauvage & Dabry de Thiersant, 1874	Cypriniformes	Cobitidae	*Cobitis* Linnaeus, 1758	11068	0.000534	2	0.20
2023	56	*Vanmanenia pingchowensis* (Fang, 1935)	Cypriniformes	Balitoridae	*Vanmanenia* Hora, 1932	11812	0.00057	2	0.20
2023	51	*Pelteobagrus eupogon* (Boulenger, 1892)	Siluriformes	Bagridae	*Pelteobagrus* Bleeker, 1864	16592	0.000801	6	0.60
2023	15	*Pelteobagrus fulvidraco* (Richardson, 1846)	Siluriformes	Bagridae	*Pelteobagrus* Bleeker, 1864	244665	0.0118	10	1.00
2023	84	*Pelteobagrus nitidus* (Sauvage & Dabry, 1874)	Siluriformes	Bagridae	*Pelteobagrus* Bleeker, 1864	75	3.62e-06	6	0.60
2023	100	*Pelteobagrus vachellii* (Richardson, 1846)	Siluriformes	Bagridae	*Pelteobagrus* Bleeker, 1864	0	0	0	0.00
2023	46	*Leiocassis crassilabris* Günther, 1864	Siluriformes	Bagridae	*Leiocassis* Bleeker, 1857	18084	0.000873	6	0.60
2023	55	*Pseudobagrus tenuis* (Günther, 1873)	Siluriformes	Bagridae	*Pseudobagrus* Bleeker, 1858	12688	0.000612	5	0.50

Year	Rank	Taxon and authority	Order	Family	Genus and authority	Reads	RRA	Sites	Frequency
2023	68	*Pseudobagrus truncatus* (Regan, 1913)	Siluriformes	Bagridae	*Pseudobagrus* Bleeker, 1858	4279	0.000206	2	0.20
2023	93	*Hemibagrus macropterus* (Bleeker, 1870)	Siluriformes	Bagridae	*Hemibagrus* Bleeker, 1862	6	2.9e-07	1	0.10
2023	78	*Liobagrus styani* Regan, 1908	Siluriformes	Amblycipitidae	*Liobagrus* Hilgendorf, 1878	562	2.71e-05	1	0.10
2023	50	*Silurus asotus* Linnaeus, 1758	Siluriformes	Siluridae	*Silurus* Linnaeus, 1758	17031	0.000822	5	0.50
2023	90	*Neosalanx taihuensis* Chen, 1956	Osmeriformes	Salangidae	*Neosalanx* Wakiya & Takahasi, 1937	20	9.65e-07	6	0.60
2023	94	*Protosalanx chinensis* (Basilewsky, 1855)	Osmeriformes	Salangidae	*Protosalanx* Regan, 1908	6	2.9e-07	2	0.20
2023	31	*Siniperca chuatsi* (Basilewsky, 1855)	Perciformes	Percichthyidae	*Siniperca* Gill, 1862	72738	0.00351	8	0.80
2023	72	*Siniperca knerii* Garman, 1912	Perciformes	Percichthyidae	*Siniperca* Gill, 1862	2149	0.000104	4	0.40
2023	92	*Siniperca roulei* Wu, 1930	Perciformes	Percichthyidae	*Siniperca* Gill, 1862	8	3.86e-07	1	0.10
2023	39	*Siniperca scherzeri* Steindachner, 1892	Perciformes	Percichthyidae	*Siniperca* Gill, 1862	42966	0.00207	3	0.30
2023	61	*Siniperca undulata* Fang & Chong, 1932	Perciformes	Percichthyidae	*Siniperca* Gill, 1862	7463	0.00036	2	0.20
2023	34	*Odontobutis sinensis* Wu, Chen & Chong, 2002	Perciformes	Odontobutidae	*Odontobutis* Bleeker, 1874	65115	0.00314	3	0.30
2023	95	*Odontobutis potamophila* (Günther, 1861)	Perciformes	Odontobutidae	*Odontobutis* Bleeker, 1874	2	9.65e-08	2	0.20
2023	65	*Micropercops swinhonis* (Günther, 1873)	Perciformes	Odontobutidae	*Micropercops* Fowler & Bean, 1920	5625	0.000271	2	0.20
2023	18	*Rhinogobius brunneus* (Temminck & Schlegel, 1845)	Perciformes	Gobiidae	*Rhinogobius* Gill, 1859	221824	0.0107	10	1.00
2023	37	*Rhinogobius cliffordpopei* (Nichols, 1925)	Perciformes	Gobiidae	*Rhinogobius* Gill, 1859	55801	0.00269	6	0.60
2023	8	*Rhinogobius giurinus* (Rutter, 1897)	Perciformes	Gobiidae	*Rhinogobius* Gill, 1859	806895	0.0389	10	1.00
2023	70	*Macropodus opercularis* (Linnaeus, 1758)	Perciformes	Osphronemidae	*Macropodus* Lacépède, 1801	2908	0.00014	2	0.20
2023	44	*Channa argus* (Cantor, 1842)	Perciformes	Channidae	*Channa* Scopoli, 1777	26844	0.0013	7	0.70
2023	62	*Micropterus salmoides* (Lacépède, 1802)	Perciformes	Centrarchidae	*Micropterus* Lacépède, 1802	7257	0.00035	3	0.30
2023	26	*Coptodon zillii* (Gervais, 1848)	Perciformes	Cichlidae	*Coptodon* Gervais, 1853	108104	0.00522	6	0.60

Year	Rank	Taxon and authority	Order	Family	Genus and authority	Reads	RRA	Sites	Frequency
2023	59	*Sinobdella sinensis* (Bleeker, 1870)	Synbranchiformes	Mastacembelidae	*Sinobdella* Kottelat & Lim, 1994	9369	0.000452	3	0.30

Table S2 (continued). 2024 campaign.

Year	Rank	Taxon and authority	Order	Family	Genus and authority	Reads	RRA	Sites	Frequency
2024	92	*Coilia brachygnathus* Kreyenberg & Pappenheim, 1908	Clupeiformes	Engraulidae	*Coilia* Gray, 1830	11	5.04e-07	1	0.10
2024	8	*Opsariichthys bidens* Günther, 1873	Cypriniformes	Cyprinidae	*Opsariichthys* Bleeker, 1863	867509	0.0397	10	1.00
2024	27	*Zacco platypus* (Temminck & Schlegel, 1846)	Cypriniformes	Cyprinidae	*Zacco* Jordan & Evermann, 1902	126411	0.00579	10	1.00
2024	60	*Rhynchocypris oxycephalus* (Sauvage & Dabry, 1874)	Cypriniformes	Cyprinidae	*Rhynchocypris* Günther, 1889	15108	0.000692	1	0.10
2024	67	*Mylopharyngodon piceus* (Richardson, 1846)	Cypriniformes	Cyprinidae	*Mylopharyngodon* Peters, 1881	9014	0.000413	10	1.00
2024	24	*Ctenopharyngodon Idella* (Valenciennes, 1844)	Cypriniformes	Cyprinidae	*Ctenopharyngodon* Steindachner, 1866	174173	0.00797	10	1.00
2024	29	*Squaliobarbus curriculus* (Richardson, 1846)	Cypriniformes	Cyprinidae	*Squaliobarbus* Günther, 1868	108085	0.00495	9	0.90
2024	78	*Ochetobius elongatus* (Kner, 1867)	Cypriniformes	Cyprinidae	*Ochetobius* Günther, 1868	1394	6.38e-05	5	0.50
2024	64	*Elopichthys Bambusa* (Richardson, 1845)	Cypriniformes	Cyprinidae	*Elopichthys* Bleeker, 1860	10558	0.000483	5	0.50
2024	89	*Sinibrama macrops* (Günther, 1868)	Cypriniformes	Cyprinidae	*Sinibrama* Wu, 1939	50	2.29e-06	6	0.60
2024	44	*Pseudolaubuca sinensis* Bleeker, 1864	Cypriniformes	Cyprinidae	*Pseudolaubuca* Bleeker, 1865	44551	0.00204	7	0.70
2024	91	*Toxabramis swinhonis* Günther, 1873	Cypriniformes	Cyprinidae	*Toxabramis* Günther, 1873	16	7.32e-07	6	0.60
2024	26	*Hemiculter bleekeri* Warpachowsky, 1887	Cypriniformes	Cyprinidae	*Hemiculter* Bleeker, 1860	138838	0.00636	9	0.90
2024	11	*Hemiculter leucisculus* (Basilewsky, 1855)	Cypriniformes	Cyprinidae	*Hemiculter* Bleeker, 1860	448503	0.0205	10	1.00
2024	22	*Hemiculterella wui* (Wang, 1935)	Cypriniformes	Cyprinidae	*Hemiculterella* Warpachowski, 1887	189927	0.00869	10	1.00
2024	47	*Pseudohemiculter dispar* (Peters, 1880)	Cypriniformes	Cyprinidae	*Pseudohemiculter* Nichols & Pope, 1927	37128	0.0017	7	0.70
2024	66	*Culter Alburnus* Basilewsky, 1855	Cypriniformes	Cyprinidae	*Culter* Basilewsky, 1855	9235	0.000423	7	0.70
2024	6	*Chanodichthys mongolicus* (Basilewsky, 1855)	Cypriniformes	Cyprinidae	*Chanodichthys* Bleeker, 1860	1070625	0.049	10	1.00
2024	18	*Chanodichthys dabryi* (Bleeker, 1871)	Cypriniformes	Cyprinidae	*Chanodichthys* Bleeker, 1860	268664	0.0123	7	0.70

Year	Rank	Taxon and authority	Order	Family	Genus and authority	Reads	RRA	Sites	Frequency
2024	4	*Cultrichthys erythropterus* (Basilewsky, 1855)	Cypriniformes	Cyprinidae	*Cultrichthys* Smith, 1938	1151511	0.0527	10	1.00
2024	73	*Megalobrama skolkovii* Dybowski, 1872	Cypriniformes	Cyprinidae	*Megalobrama* Dybowski, 1872	2886	0.000132	2	0.20
2024	61	*Megalobrama amblycephala* Yih, 1955	Cypriniformes	Cyprinidae	*Megalobrama* Dybowski, 1872	13961	0.000639	10	1.00
2024	31	*Parabramis pekinensis* (Basilewsky, 1855)	Cypriniformes	Cyprinidae	*Parabramis* Bleeker, 1864	104425	0.00478	10	1.00
2024	1	*Xenocypris argentea* Günther, 1868	Cypriniformes	Cyprinidae	*Xenocypris* Günther, 1868	5470760	0.25	10	1.00
2024	5	*Xenocypris davidi* Bleeker, 1871	Cypriniformes	Cyprinidae	*Xenocypris* Günther, 1868	1119629	0.0513	10	1.00
2024	2	*Xenocypris microlepis* Bleeker, 1871	Cypriniformes	Cyprinidae	*Xenocypris* Günther, 1868	2311426	0.106	10	1.00
2024	3	*Distoechodon tumirostris* Peters, 1880	Cypriniformes	Cyprinidae	*Distoechodon* Peters, 1880	1319032	0.0604	10	1.00
2024	25	*Pseudobrama simoni* (Bleeker, 1864)	Cypriniformes	Cyprinidae	*Pseudobrama* Bleeker, 1871	163438	0.00748	7	0.70
2024	49	*Hypophthalmichthys molitrix* (Valenciennes, 1844)	Cypriniformes	Cyprinidae	*Hypophthalmichthys* Bleeker, 1860	30054	0.00138	10	1.00
2024	33	*Aristichthys nobilis* (Richardson, 1845)	Cypriniformes	Cyprinidae	*Aristichthys* Oshima, 1919	97349	0.00446	9	0.90
2024	21	*Rhodeus ocellatus* (Kner, 1867)	Cypriniformes	Cyprinidae	*Rhodeus* Agassiz, 1835	224097	0.0103	9	0.90
2024	53	*Rhodeus sinensis* Günther, 1868	Cypriniformes	Cyprinidae	*Rhodeus* Agassiz, 1835	23241	0.00106	10	1.00
2024	77	*Acheilognathus barbatulus* Günther, 1873	Cypriniformes	Cyprinidae	*Acheilognathus* Bleeker, 1860	2013	9.21e-05	3	0.30
2024	45	*Acheilognathus barbatus* Nichols, 1926	Cypriniformes	Cyprinidae	*Acheilognathus* Bleeker, 1860	42640	0.00195	7	0.70
2024	56	*Acheilognathus chankaensis* (Dybowski, 1872)	Cypriniformes	Cyprinidae	*Acheilognathus* Bleeker, 1860	20576	0.000942	6	0.60
2024	51	*Acheilognathus hypselonotus* (Bleeker, 1871)	Cypriniformes	Cyprinidae	*Acheilognathus* Bleeker, 1860	25755	0.00118	5	0.50
2024	48	*Acheilognathus imberbis* Günther, 1868	Cypriniformes	Cyprinidae	*Acheilognathus* Bleeker, 1860	34322	0.00157	8	0.80
2024	83	*Acheilognathus macropterus* (Bleeker, 1871)	Cypriniformes	Cyprinidae	*Acheilognathus* Bleeker, 1860	252	1.15e-05	4	0.40
2024	15	*Acheilognathus rhombeus* (Temminck & Schlegel, 1846)	Cypriniformes	Cyprinidae	*Acheilognathus* Bleeker, 1860	283562	0.013	9	0.90

Year	Rank	Taxon and authority	Order	Family	Genus and authority	Reads	RRA	Sites	Frequency
2024	86	*Acheilognathus tonkinensis* (Vaillant, 1892)	Cypriniformes	Cyprinidae	*Acheilognathus* Bleeker, 1860	131	6e-06	3	0.30
2024	65	*Hemibarbus labeo* (Pallas, 1776)	Cypriniformes	Cyprinidae	*Hemibarbus* Bleeker, 1860	9605	0.00044	7	0.70
2024	37	*Hemibarbus maculatus* Bleeker, 1871	Cypriniformes	Cyprinidae	*Hemibarbus* Bleeker, 1860	77381	0.00354	9	0.90
2024	79	*Pseudorasbora elongata* Wu, 1939	Cypriniformes	Cyprinidae	*Pseudorasbora* Bleeker, 1860	1183	5.42e-05	1	0.10
2024	50	*Pseudorasbora parva* (Temminck & Schlegel, 1846)	Cypriniformes	Cyprinidae	*Pseudorasbora* Bleeker, 1860	29848	0.00137	7	0.70
2024	69	*Sarcocheilichthys nigripinnis* (Günther, 1873)	Cypriniformes	Cyprinidae	*Sarcocheilichthys* Bleeker, 1860	5474	0.000251	3	0.30
2024	62	*Sarcocheilichthys parvus* Nichols, 1930	Cypriniformes	Cyprinidae	*Sarcocheilichthys* Bleeker, 1860	12084	0.000553	5	0.50
2024	90	*Sarcocheilichthys kiangsiensis* Nichols, 1930	Cypriniformes	Cyprinidae	*Sarcocheilichthys* Bleeker, 1860	17	7.78e-07	4	0.40
2024	52	*Sarcocheilichthys sinensis* Bleeker, 1871	Cypriniformes	Cyprinidae	*Sarcocheilichthys* Bleeker, 1860	23506	0.00108	6	0.60
2024	23	*Gnathopogon imberbis* (Sauvage & Dabry, 1874)	Cypriniformes	Cyprinidae	*Gnathopogon* Bleeker, 1860	180789	0.00828	10	1.00
2024	13	*Squalidus argentatus* (Sauvage & Dabry, 1874)	Cypriniformes	Cyprinidae	*Squalidus* Dybowski, 1872	358133	0.0164	10	1.00
2024	82	*Abbottina rivularis* (Basilewsky, 1855)	Cypriniformes	Cyprinidae	*Abbottina* Jordan & Fowler, 1903	309	1.41e-05	4	0.40
2024	16	*Microphysogobio fukiensis* (Nichols, 1926)	Cypriniformes	Cyprinidae	*Microphysogobio* Mori, 1933	279117	0.0128	9	0.90
2024	41	*Pseudogobio vaillanti* (Sauvage, 1878)	Cypriniformes	Cyprinidae	*Pseudogobio* Bleeker, 1860	59818	0.00274	8	0.80
2024	28	*Saurogobio dabryi* Bleeker, 1871	Cypriniformes	Cyprinidae	*Saurogobio* Bleeker, 1870	123053	0.00563	9	0.90
2024	84	*Saurogobio dumerili* Bleeker, 1871	Cypriniformes	Cyprinidae	*Saurogobio* Bleeker, 1870	196	8.97e-06	2	0.20
2024	55	*Gobiobotia filifer* (Garman, 1912)	Cypriniformes	Cyprinidae	*Gobiobotia* Kreyenberg, 1911	21223	0.000972	8	0.80
2024	85	*Gobiobotia meridionalis* Chen & Tsao, 1982	Cypriniformes	Cyprinidae	*Gobiobotia* Kreyenberg, 1911	152	6.96e-06	1	0.10
2024	32	*Spinibarbus hollandi* Oshima, 1919	Cypriniformes	Cyprinidae	*Spinibarbus* Oshima, 1919	103610	0.00474	6	0.60
2024	17	*Acrossocheilus kreyenbergii* (Regan, 1908)	Cypriniformes	Cyprinidae	*Acrossocheilus* Oshima, 1919	274392	0.0126	9	0.90

Year	Rank	Taxon and authority	Order	Family	Genus and authority	Reads	RRA	Sites	Frequency
2024	72	*Cirrhinus molitorella* (Valenciennes, 1844)	Cypriniformes	Cyprinidae	*Cirrhinus* Oken, 1817	3216	0.000147	4	0.40
2024	95	*Cirrhinus mrigala* (Hamilton, 1822)	Cypriniformes	Cyprinidae	*Cirrhinus* Oken, 1817	0	0	0	0.00
2024	7	*Cyprinus carpio* Linnaeus, 1758	Cypriniformes	Cyprinidae	*Cyprinus* Linnaeus, 1758	887537	0.0406	10	1.00
2024	9	*Carassius auratus* (Linnaeus, 1758)	Cypriniformes	Cyprinidae	*Carassius* Jarocki, 1822	759247	0.0348	10	1.00
2024	81	*Parabotia banarescui* (Nalbant, 1965)	Cypriniformes	Cobitidae	*Parabotia* Dabry de Thiersant, 1872	404	1.85e-05	7	0.70
2024	42	*Parabotia fasciata* Dabry de Thiersant, 1872	Cypriniformes	Cobitidae	*Parabotia* Dabry de Thiersant, 1872	55156	0.00252	8	0.80
2024	87	*Parabotia kiangsiensis* Liu & Guo, 1986	Cypriniformes	Cobitidae	*Parabotia* Dabry de Thiersant, 1872	116	5.31e-06	4	0.40
2024	80	*Parabotia lijiangensis* Chen, 1980	Cypriniformes	Cobitidae	*Parabotia* Dabry de Thiersant, 1872	783	3.58e-05	6	0.60
2024	59	*Misgurnus anguillicaudatus* (Cantor, 1842)	Cypriniformes	Cobitidae	*Misgurnus* Lacépède, 1803	15549	0.000712	3	0.30
2024	70	*Paramisgurnus dabryanus* Dabry de Thiersant, 1872	Cypriniformes	Cobitidae	*Paramisgurnus* Dabry de Thiersant, 1872	4403	0.000202	3	0.30
2024	57	*Leptobotia tientainensis* (Wu, 1930)	Cypriniformes	Cobitidae	*Leptobotia* Bleeker, 1870	20358	0.000932	8	0.80
2024	14	*Cobitis sinensis* Sauvage & Dabry de Thiersant, 1874	Cypriniformes	Cobitidae	*Cobitis* Linnaeus, 1758	314070	0.0144	8	0.80
2024	12	*Vanmanenia pingchowensis* (Fang, 1935)	Cypriniformes	Balitoridae	*Vanmanenia* Hora, 1932	371145	0.017	4	0.40
2024	88	*Pelteobagrus eupogon* (Boulenger, 1892)	Siluriformes	Bagridae	*Pelteobagrus* Bleeker, 1864	89	4.07e-06	6	0.60
2024	19	*Pelteobagrus fulvidraco* (Richardson, 1846)	Siluriformes	Bagridae	*Pelteobagrus* Bleeker, 1864	263687	0.0121	9	0.90
2024	71	*Pelteobagrus nitidus* (Sauvage & Dabry, 1874)	Siluriformes	Bagridae	*Pelteobagrus* Bleeker, 1864	3747	0.000172	7	0.70
2024	30	*Pelteobagrus vachellii* (Richardson, 1846)	Siluriformes	Bagridae	*Pelteobagrus* Bleeker, 1864	107953	0.00494	4	0.40
2024	39	*Leiocassis crassilabris* Günther, 1864	Siluriformes	Bagridae	*Leiocassis* Bleeker, 1857	74492	0.00341	5	0.50
2024	100	*Pseudobagrus tenuis* (Günther, 1873)	Siluriformes	Bagridae	*Pseudobagrus* Bleeker, 1858	0	0	0	0.00
2024	94	*Pseudobagrus truncatus* (Regan, 1913)	Siluriformes	Bagridae	*Pseudobagrus* Bleeker, 1858	4	1.83e-07	1	0.10

Year	Rank	Taxon and authority	Order	Family	Genus and authority	Reads	RRA	Sites	Frequency
2024	96	*Hemibagrus macropterus* (Bleeker, 1870)	Siluriformes	Bagridae	*Hemibagrus* Bleeker, 1862	0	0	0	0.00
2024	63	*Liobagrus styani* Regan, 1908	Siluriformes	Amblycipitidae	*Liobagrus* Hilgendorf, 1878	10662	0.000488	2	0.20
2024	38	*Silurus asotus* Linnaeus, 1758	Siluriformes	Siluridae	*Silurus* Linnaeus, 1758	75728	0.00347	9	0.90
2024	74	*Neosalanx taihuensis* Chen, 1956	Osmeriformes	Salangidae	*Neosalanx* Wakiya & Takahasi, 1937	2760	0.000126	1	0.10
2024	99	*Protosalanx chinensis* (Basilewsky, 1855)	Osmeriformes	Salangidae	*Protosalanx* Regan, 1908	0	0	0	0.00
2024	36	*Siniperca chuatsi* (Basilewsky, 1855)	Perciformes	Percichthyidae	*Siniperca* Gill, 1862	89389	0.00409	10	1.00
2024	75	*Siniperca knerii* Garman, 1912	Perciformes	Percichthyidae	*Siniperca* Gill, 1862	2554	0.000117	6	0.60
2024	93	*Siniperca roulei* Wu, 1930	Perciformes	Percichthyidae	*Siniperca* Gill, 1862	8	3.66e-07	1	0.10
2024	58	*Siniperca scherzeri* Steindachner, 1892	Perciformes	Percichthyidae	*Siniperca* Gill, 1862	20180	0.000924	5	0.50
2024	46	*Siniperca undulata* Fang & Chong, 1932	Perciformes	Percichthyidae	*Siniperca* Gill, 1862	39010	0.00179	6	0.60
2024	34	*Odontobutis sinensis* Wu, Chen & Chong, 2002	Perciformes	Odontobutidae	*Odontobutis* Bleeker, 1874	93554	0.00428	6	0.60
2024	98	*Odontobutis potamophila* (Günther, 1861)	Perciformes	Odontobutidae	*Odontobutis* Bleeker, 1874	0	0	0	0.00
2024	76	*Micropercops swinhonis* (Günther, 1873)	Perciformes	Odontobutidae	*Micropercops* Fowler & Bean, 1920	2218	0.000102	1	0.10
2024	35	*Rhinogobius brunneus* (Temminck & Schlegel, 1845)	Perciformes	Gobiidae	*Rhinogobius* Gill, 1859	93121	0.00426	8	0.80
2024	20	*Rhinogobius cliffordpopei* (Nichols, 1925)	Perciformes	Gobiidae	*Rhinogobius* Gill, 1859	252478	0.0116	8	0.80
2024	10	*Rhinogobius giurinus* (Rutter, 1897)	Perciformes	Gobiidae	*Rhinogobius* Gill, 1859	620718	0.0284	10	1.00
2024	97	*Macropodus opercularis* (Linnaeus, 1758)	Perciformes	Osphronemidae	*Macropodus* Lacépède, 1801	0	0	0	0.00
2024	68	*Channa argus* (Cantor, 1842)	Perciformes	Channidae	*Channa* Scopoli, 1777	8681	0.000397	2	0.20
2024	54	*Micropterus salmoides* (Lacépède, 1802)	Perciformes	Centrarchidae	*Micropterus* Lacépède, 1802	22772	0.00104	5	0.50
2024	40	*Coptodon zillii* (Gervais, 1848)	Perciformes	Cichlidae	*Coptodon* Gervais, 1853	63893	0.00292	10	1.00
2024	43	*Sinobdella sinensis* (Bleeker, 1870)	Synbranchiformes	Mastacembelidae	*Sinobdella* Kottelat & Lim, 1994	44799	0.00205	5	0.50

Note: zero-RRA records are retained. RRA is a proportion, and occurrence frequency is the number of detected sites divided by ten.

Table S3. RDA screening and constrained-model results.

VIF screening

Screening step	Environmental variable	VIF	Retained
1	Water temperature	4.675	Yes
1	pH	14.606	Yes
1	Dissolved oxygen	17.802	Yes
1	Permanganate index	68.114	Yes
1	Total nitrogen	13.203	Yes
1	Total phosphorus	11.426	Yes
1	Ammonia nitrogen	19.431	Yes
1	Petroleum	75.610	No
1	Volatile phenol	6.098	Yes
1	Copper	25.925	Yes
1	Transparency	3.941	Yes
1	Chlorophyll a	14.377	Yes
2	Water temperature	4.239	Yes
2	pH	13.215	Yes
2	Dissolved oxygen	17.741	Yes
2	Permanganate index	23.400	Yes
2	Total nitrogen	12.904	Yes
2	Total phosphorus	8.859	Yes
2	Ammonia nitrogen	16.246	Yes
2	Volatile phenol	4.556	Yes
2	Copper	25.845	No
2	Transparency	3.354	Yes
2	Chlorophyll a	11.710	Yes
3	Water temperature	3.401	Yes
3	pH	13.214	Yes
3	Dissolved oxygen	17.740	Yes
3	Permanganate index	20.277	No
3	Total nitrogen	9.648	Yes
3	Total phosphorus	3.389	Yes
3	Ammonia nitrogen	14.348	Yes
3	Volatile phenol	3.700	Yes
3	Transparency	3.297	Yes
3	Chlorophyll a	5.271	Yes
4	Water temperature	3.373	Yes
4	pH	11.952	Yes
4	Dissolved oxygen	5.424	Yes
4	Total nitrogen	8.777	Yes
4	Total phosphorus	2.906	Yes
4	Ammonia nitrogen	12.158	No

Screening step	Environmental variable	VIF	Retained
4	Volatile phenol	3.195	Yes
4	Transparency	2.874	Yes
4	Chlorophyll a	3.821	Yes
5	Water temperature	2.739	Yes
5	pH	3.890	Yes
5	Dissolved oxygen	5.207	Yes
5	Total nitrogen	6.067	Yes
5	Total phosphorus	2.869	Yes
5	Volatile phenol	2.730	Yes
5	Transparency	2.193	Yes
5	Chlorophyll a	3.774	Yes

Selected variables

Environmental variable
Water temperature
pH
Dissolved oxygen
Total nitrogen
Total phosphorus
Volatile phenol
Transparency
Chlorophyll a

Overall constrained models

R^2 values are relative to total community variation. Tests use 1,023 site-restricted reduced-model residual permutations. The eight-variable model conditioned on site and campaign has only one residual degree of freedom and is not used for ecological inference.

Model	df	Residual df	F	p	R^2	Adjusted R^2
Two environmental PCs; site + campaign	2	7	0.971	0.420	0.068	-0.004
Eight variables; campaign only (diagnostic)	8	10	1.192	0.394	0.361	0.062
Eight variables; site + campaign (diagnostic)	8	1	0.886	0.486	0.273	-0.074

Single-variable partial RDA conditioned on site and campaign

Variable	df	Residual df	F	p	BH-adjusted p
Water temperature	1	8	0.513	0.945	0.945
pH	1	8	1.007	0.391	0.521
Dissolved oxygen	1	8	1.340	0.264	0.521
Total nitrogen	1	8	1.153	0.332	0.521

Variable	df	Residual df	F	p	BH-adjusted p
Total phosphorus	1	8	0.962	0.369	0.521
Volatile phenol	1	8	1.478	0.055	0.438
Transparency	1	8	1.176	0.500	0.571
Chlorophyll *a*	1	8	1.662	0.209	0.521

Table S3 (continued). Loadings of the two environmental principal components.

Environmental variable	PC1	PC2
Water temperature	-0.040	0.357
pH	0.415	0.509
Dissolved oxygen	0.268	-0.113
Total nitrogen	-0.425	0.413
Total phosphorus	0.186	0.620
Volatile phenol	-0.214	0.130
Transparency	-0.432	0.166
Chlorophyll *a*	-0.554	0.013

Table S4. Full alpha-diversity, beta-diversity (PERMANOVA), and PERMDISP outputs.

Paired alpha-diversity tests (ten pairs; change = 2024 minus 2023)

Metric	t (df = 9)	Mean change	95% CI	p	BH p	Shapiro p
Observed richness	0.697	1.800	-4.039 to 7.639	0.503	0.503	0.191
Shannon	1.045	0.126	-0.146 to 0.398	0.323	0.420	0.169
Simpson	1.530	0.027	-0.013 to 0.067	0.160	0.420	0.150
Pielou evenness	1.107	0.027	-0.028 to 0.083	0.297	0.420	0.254
Rarefied richness	1.017	2.690	-3.294 to 8.675	0.336	0.420	0.349

Beta-diversity and dispersion tests

Campaign tests enumerate 1,023 within-site exchanges; river tests use 9,999 permutations within a campaign. BH correction is applied separately to campaign and river comparisons and separately to PERMANOVA and PERMDISP. PERMDISP uses group medians.

Distance	Test	Comparison	df	F	R^2	p	BH p
Bray-Curtis	PERMANOVA	campaign	1, 18	7.466	0.293	0.002	0.002
Bray-Curtis	PERMDISP	campaign	1, 18	0.009	—	0.907	0.907
Bray-Curtis	PERMANOVA	river 2023	2, 7	2.550	0.421	0.021	0.042
Bray-Curtis	PERMDISP	river 2023	2, 7	0.387	—	0.662	0.881
Bray-Curtis	PERMANOVA	river 2024	2, 7	1.568	0.309	0.074	0.074
Bray-Curtis	PERMDISP	river 2024	2, 7	0.345	—	0.695	0.881
Jaccard	PERMANOVA	campaign	1, 18	4.095	0.185	0.002	0.002
Jaccard	PERMDISP	campaign	1, 18	0.051	—	0.782	0.907
Jaccard	PERMANOVA	river 2023	2, 7	2.261	0.392	0.005	0.018
Jaccard	PERMDISP	river 2023	2, 7	1.571	—	0.279	0.881
Jaccard	PERMANOVA	river 2024	2, 7	1.743	0.333	0.038	0.051
Jaccard	PERMDISP	river 2024	2, 7	0.151	—	0.881	0.881

Table S4 (continued). River-group alpha-diversity tests (ten sites per campaign; df = 2). Rank-based $\eta^2H = (H - k + 1)/(n - k)$, with $k = 3$ and $n = 10$; negative estimates are reported without truncation.

Campaign	Metric	H	η^2H	p	BH p
2023	Observed richness	1.333	-0.095	0.513	0.642
2023	Shannon	5.982	0.569	0.050	0.251
2023	Simpson	4.818	0.403	0.090	0.300
2023	Pielou evenness	5.982	0.569	0.050	0.251
2023	Rarefied richness	3.073	0.153	0.215	0.380
2024	Observed richness	0.764	-0.177	0.683	0.683
2024	Shannon	1.655	-0.049	0.437	0.625
2024	Simpson	3.391	0.199	0.184	0.380
2024	Pielou evenness	2.955	0.136	0.228	0.380
2024	Rarefied richness	1.064	-0.134	0.588	0.653

Table S4 (continued). Site-level paired alpha-diversity data.

Campaign	Site	Richness	Rarefied richness	Shannon	Simpson	Pielou
2023	A1	61	56.244	2.844	0.918	0.692
2023	A2	74	69.425	2.999	0.924	0.697
2023	A3	62	59.773	2.617	0.876	0.634
2023	B1	49	48.972	2.109	0.778	0.542
2023	B2	60	51.977	1.931	0.800	0.472
2023	B3	66	62.050	2.429	0.872	0.580
2023	B4	63	58.285	2.085	0.789	0.503
2023	C1	55	50.069	1.826	0.749	0.456
2023	C2	63	57.658	2.536	0.877	0.612
2023	C3	51	44.004	1.405	0.637	0.357
2024	A1	59	56.463	2.622	0.892	0.643
2024	A2	65	61.661	2.868	0.907	0.687

Campaign	Site	Richness	Rarefied richness	Shannon	Simpson	Pielou
2024	A3	65	61.343	2.452	0.864	0.587
2024	B1	43	43.000	1.817	0.749	0.483
2024	B2	74	70.253	2.722	0.861	0.632
2024	B3	65	59.364	2.432	0.874	0.583
2024	B4	75	71.348	2.514	0.835	0.582
2024	C1	67	59.621	2.499	0.877	0.594
2024	C2	62	58.454	2.568	0.889	0.622
2024	C3	47	43.855	1.544	0.741	0.401

Table S5. The ten taxa with the highest combined RRA and their 80 campaign-adjusted partial-rank correlations with eight environmental variables.

Top-taxon selection

Taxon	Combined RRA	Mean RRA	Detected units (of 20)	Rank by combined RRA
Xenocypris microlepis	3.7375	0.1869	20	1
Xenocypris argentea	2.9821	0.1491	20	2
Aristichthys nobilis	1.1066	0.0553	19	3
Distoechodon tumirostris	0.9487	0.0474	20	4
Cyprinus carpio	0.9216	0.0461	20	5
Sarcocheilichthys sinensis	0.8740	0.0437	16	6
Rhinogobius giurinus	0.8161	0.0408	20	7
Xenocypris davidi	0.5769	0.0288	20	8
Chanodichthys mongolicus	0.5572	0.0279	20	9
Sarcocheilichthys kiangsiensis	0.5452	0.0273	14	10

Campaign-adjusted correlations

Taxon	Environmental variable	Partial-rank r (20)	Approx. 95% CI
Xenocypris microlepis	Water temperature	0.185	-0.166 to 0.534
Xenocypris microlepis	pH	0.070	-0.418 to 0.482
Xenocypris microlepis	Dissolved oxygen	0.150	-0.192 to 0.507
Xenocypris microlepis	Total nitrogen	0.107	-0.412 to 0.583

Taxon	Environmental variable	Partial-rank r (20)	Approx. 95% CI
Xenocypris microlepis	Total phosphorus	0.160	-0.327 to 0.496
Xenocypris microlepis	Volatile phenol	0.505	0.233 to 0.631
Xenocypris microlepis	Transparency	-0.405	-0.756 to 0.012
Xenocypris microlepis	Chlorophyll a	-0.447	-0.813 to 0.111
Xenocypris argentea	Water temperature	-0.045	-0.541 to 0.450
Xenocypris argentea	pH	0.347	-0.111 to 0.701
Xenocypris argentea	Dissolved oxygen	-0.135	-0.647 to 0.567
Xenocypris argentea	Total nitrogen	0.394	-0.263 to 0.742
Xenocypris argentea	Total phosphorus	0.412	0.011 to 0.755
Xenocypris argentea	Volatile phenol	0.241	-0.294 to 0.609
Xenocypris argentea	Transparency	-0.172	-0.720 to 0.300
Xenocypris argentea	Chlorophyll a	-0.082	-0.509 to 0.348
Aristichthys nobilis	Water temperature	0.195	-0.181 to 0.466
Aristichthys nobilis	pH	0.074	-0.233 to 0.346
Aristichthys nobilis	Dissolved oxygen	0.045	-0.499 to 0.548
Aristichthys nobilis	Total nitrogen	-0.016	-0.579 to 0.524
Aristichthys nobilis	Total phosphorus	-0.199	-0.586 to 0.208
Aristichthys nobilis	Volatile phenol	0.556	0.324 to 0.662
Aristichthys nobilis	Transparency	-0.256	-0.700 to 0.414
Aristichthys nobilis	Chlorophyll a	-0.298	-0.576 to 0.255
Distoechodon tumirostris	Water temperature	-0.097	-0.619 to 0.366
Distoechodon tumirostris	pH	-0.190	-0.523 to 0.154
Distoechodon tumirostris	Dissolved oxygen	-0.065	-0.513 to 0.332
Distoechodon tumirostris	Total nitrogen	-0.306	-0.668 to 0.065
Distoechodon tumirostris	Total phosphorus	-0.254	-0.659 to 0.231
Distoechodon tumirostris	Volatile phenol	-0.169	-0.531 to 0.262
Distoechodon tumirostris	Transparency	-0.169	-0.623 to 0.356
Distoechodon tumirostris	Chlorophyll a	-0.061	-0.425 to 0.274
Cyprinus carpio	Water temperature	-0.105	-0.756 to 0.508
Cyprinus carpio	pH	0.026	-0.532 to 0.548
Cyprinus carpio	Dissolved oxygen	-0.306	-0.714 to 0.101
Cyprinus carpio	Total nitrogen	0.129	-0.356 to 0.529
Cyprinus carpio	Total phosphorus	0.238	-0.166 to 0.554
Cyprinus carpio	Volatile phenol	-0.246	-0.634 to 0.253
Cyprinus carpio	Transparency	-0.146	-0.595 to 0.358
Cyprinus carpio	Chlorophyll a	-0.017	-0.509 to 0.545
Sarcocheilichthys sinensis	Water temperature	0.176	-0.350 to 0.550
Sarcocheilichthys sinensis	pH	-0.169	-0.631 to 0.305
Sarcocheilichthys sinensis	Dissolved oxygen	-0.062	-0.412 to 0.368
Sarcocheilichthys sinensis	Total nitrogen	-0.011	-0.397 to 0.535
Sarcocheilichthys sinensis	Total phosphorus	-0.176	-0.684 to 0.415
Sarcocheilichthys sinensis	Volatile phenol	-0.485	-0.679 to -0.090
Sarcocheilichthys sinensis	Transparency	-0.014	-0.327 to 0.268

Taxon	Environmental variable	Partial-rank r (20)	Approx. 95% CI
Sarcocheilichthys sinensis	Chlorophyll a	0.399	-0.049 to 0.731
Rhinogobius giurinus	Water temperature	0.141	-0.409 to 0.572
Rhinogobius giurinus	pH	-0.027	-0.460 to 0.373
Rhinogobius giurinus	Dissolved oxygen	-0.367	-0.799 to 0.275
Rhinogobius giurinus	Total nitrogen	0.020	-0.450 to 0.436
Rhinogobius giurinus	Total phosphorus	-0.271	-0.704 to 0.413
Rhinogobius giurinus	Volatile phenol	-0.219	-0.672 to 0.217
Rhinogobius giurinus	Transparency	0.365	-0.096 to 0.699
Rhinogobius giurinus	Chlorophyll a	0.312	-0.042 to 0.623
Xenocypris davidi	Water temperature	0.259	-0.368 to 0.693
Xenocypris davidi	pH	0.301	-0.266 to 0.664
Xenocypris davidi	Dissolved oxygen	0.122	-0.533 to 0.631
Xenocypris davidi	Total nitrogen	0.094	-0.256 to 0.600
Xenocypris davidi	Total phosphorus	0.208	-0.414 to 0.672
Xenocypris davidi	Volatile phenol	0.118	-0.321 to 0.600
Xenocypris davidi	Transparency	-0.349	-0.776 to 0.179
Xenocypris davidi	Chlorophyll a	-0.354	-0.625 to 0.047
Chanodichthys mongolicus	Water temperature	-0.118	-0.660 to 0.377
Chanodichthys mongolicus	pH	-0.363	-0.752 to 0.033
Chanodichthys mongolicus	Dissolved oxygen	-0.171	-0.710 to 0.491
Chanodichthys mongolicus	Total nitrogen	0.052	-0.293 to 0.396
Chanodichthys mongolicus	Total phosphorus	0.068	-0.286 to 0.361
Chanodichthys mongolicus	Volatile phenol	-0.164	-0.555 to 0.213
Chanodichthys mongolicus	Transparency	-0.004	-0.477 to 0.454
Chanodichthys mongolicus	Chlorophyll a	0.121	-0.486 to 0.597
Sarcocheilichthys kiangsiensis	Water temperature	-0.323	-0.656 to 0.090
Sarcocheilichthys kiangsiensis	pH	-0.216	-0.682 to 0.286
Sarcocheilichthys kiangsiensis	Dissolved oxygen	0.077	-0.340 to 0.462
Sarcocheilichthys kiangsiensis	Total nitrogen	-0.283	-0.605 to 0.138
Sarcocheilichthys kiangsiensis	Total phosphorus	0.114	-0.336 to 0.592
Sarcocheilichthys kiangsiensis	Volatile phenol	-0.613	-0.724 to -0.388
Sarcocheilichthys kiangsiensis	Transparency	0.053	-0.484 to 0.553
Sarcocheilichthys kiangsiensis	Chlorophyll a	0.205	-0.206 to 0.584

Note: Correlations use all 20 site-by-campaign observations. Approximate pointwise 95% confidence intervals come from 3,999 bootstrap resamples of the ten sites, retaining each site's paired observations. The intervals are not adjusted for the 80 comparisons and are presented descriptively.

Table S6. Read depth and richness of the 60 technical process replicates.

Sample	Fish reads	Richness	Depth / campaign median	Low depth
Y2023_A1-1	632,349	48	0.880	No
Y2023_A1-2	786,335	47	1.094	No
Y2023_A1-3	643,353	46	0.895	No
Y2023_A2-1	744,101	65	1.035	No
Y2023_A2-2	717,783	52	0.999	No
Y2023_A2-3	845,203	53	1.176	No
Y2023_A3-1	497,587	21	0.692	No
Y2023_A3-2	472,765	52	0.658	No
Y2023_A3-3	502,274	49	0.699	No
Y2023_B1-1	13,351	19	0.019	Yes
Y2023_B1-2	408,754	36	0.569	No
Y2023_B1-3	453,498	33	0.631	No
Y2023_B2-1	845,680	33	1.177	No
Y2023_B2-2	732,573	43	1.019	No
Y2023_B2-3	690,924	48	0.961	No
Y2023_B3-1	513,010	53	0.714	No
Y2023_B3-2	497,631	43	0.692	No
Y2023_B3-3	538,074	37	0.749	No
Y2023_B4-1	700,249	48	0.974	No
Y2023_B4-2	736,114	46	1.024	No
Y2023_B4-3	719,530	35	1.001	No
Y2023_C1-1	888,938	49	1.237	No
Y2023_C1-2	631,544	29	0.879	No
Y2023_C1-3	1,012,105	26	1.408	No
Y2023_C2-1	1,108,233	49	1.542	No
Y2023_C2-2	753,656	43	1.049	No
Y2023_C2-3	818,562	39	1.139	No
Y2023_C3-1	1,061,718	30	1.477	No
Y2023_C3-2	929,256	37	1.293	No
Y2023_C3-3	829,738	33	1.155	No
Y2024_A1-1	721,132	54	0.986	No
Y2024_A1-2	893,470	55	1.221	No
Y2024_A1-3	709,441	54	0.970	No
Y2024_A2-1	1,037,290	58	1.418	No
Y2024_A2-2	1,014,682	60	1.387	No
Y2024_A2-3	976,109	63	1.334	No
Y2024_A3-1	667,776	61	0.913	No
Y2024_A3-2	745,516	57	1.019	No
Y2024_A3-3	574,438	42	0.785	No
Y2024_B1-1	438,741	35	0.600	No

Sample	Fish reads	Richness	Depth / campaign median	Low depth
Y2024_B1-2	527	25	<0.001	Yes
Y2024_B1-3	433,655	23	0.593	No
Y2024_B2-1	968,544	68	1.324	No
Y2024_B2-2	746,519	53	1.020	No
Y2024_B2-3	748,099	69	1.022	No
Y2024_B3-1	973,464	51	1.330	No
Y2024_B3-2	1,179,019	51	1.611	No
Y2024_B3-3	704,299	56	0.963	No
Y2024_B4-1	882,937	58	1.207	No
Y2024_B4-2	860,624	59	1.176	No
Y2024_B4-3	742,300	64	1.014	No
Y2024_C1-1	707,630	54	0.967	No
Y2024_C1-2	554,870	52	0.758	No
Y2024_C1-3	801,589	48	1.095	No
Y2024_C2-1	571,429	54	0.781	No
Y2024_C2-2	559,980	54	0.765	No
Y2024_C2-3	651,515	50	0.890	No
Y2024_C3-1	624,911	37	0.854	No
Y2024_C3-2	783,452	40	1.071	No
Y2024_C3-3	571,273	41	0.781	No

Note: Low depth indicates fewer than 10% of the campaign median reads.

Table S6 (continued). Within-site, within-campaign technical-replicate Bray–Curtis dissimilarity.

Campaign	Site	Mean dissimilarity	Maximum dissimilarity
2023	A1	0.295	0.317
2023	A2	0.201	0.235
2023	A3	0.422	0.542
2023	B1	0.305	0.381
2023	B2	0.388	0.574
2023	B3	0.389	0.442
2023	B4	0.219	0.257
2023	C1	0.542	0.801
2023	C2	0.263	0.301
2023	C3	0.118	0.160
2024	A1	0.131	0.179
2024	A2	0.162	0.198
2024	A3	0.350	0.501
2024	B1	0.193	0.278
2024	B2	0.196	0.281
2024	B3	0.510	0.668

Campaign	Site	Mean dissimilarity	Maximum dissimilarity
2024	B4	0.294	0.370
2024	C1	0.267	0.372
2024	C2	0.200	0.234
2024	C3	0.209	0.285

Table S7. Low-read sensitivity analysis. Primary data are unchanged; thresholds are applied to fish-taxon counts.

Scenario	Total taxa	2023	2024	Shared	Reads retained (%)
Primary data	100	96	94	90	100.00000
≤1, before pooling	99	95	94	90	99.99919
≤2, before pooling	99	94	94	89	99.99840
<5, before pooling	99	94	92	87	99.99710
≤5, before pooling	98	93	92	87	99.99660
≤10, before pooling	96	89	90	83	99.99369
<5, after pooling	99	94	93	88	99.99884
≤5, after pooling	98	93	93	88	99.99865
≤10, after pooling	96	89	90	83	99.99755

Table S7 (continued). Read-sum versus equal-weight RRA profiles.

Campaign	Site	Bray–Curtis difference	Spearman ρ	Maximum taxon RRA difference
2023	A1	0.0131	0.9998	0.0029
2023	A2	0.0063	0.9999	0.0012
2023	A3	0.0026	0.9999	<0.001
2023	B1	0.1127	0.9942	0.0725
2023	B2	0.0222	0.9996	0.0101
2023	B3	0.0040	0.9998	<0.001
2023	B4	0.0021	0.9999	<0.001

Campaign	Site	Bray–Curtis difference	Spearman ρ	Maximum taxon RRA difference
2023	C1	0.0126	0.9997	0.0064
2023	C2	0.0230	0.9996	0.0050
2023	C3	0.0068	0.9997	0.0023
2024	A1	0.0032	0.9999	<0.001
2024	A2	0.0017	1.0000	<0.001
2024	A3	0.0222	0.9998	0.0081
2024	B1	0.0906	0.9900	0.0336
2024	B2	0.0083	0.9997	0.0035
2024	B3	0.0560	0.9992	0.0209
2024	B4	0.0068	0.9996	0.0022
2024	C1	0.0208	0.9996	0.0053
2024	C2	0.0066	0.9998	0.0025
2024	C3	0.0050	0.9999	0.0035

Table S7 (continued). Main sensitivity results. Both campaign PERMANOVA metrics remain significant in every low-read scenario (BH-adjusted p = 0.001953).

Pooling method	Bray–Curtis R^2	Campaign p
Read-sum pooling	0.2932	0.002
Equal-weight replicate RRA	0.2986	0.002

Table S8. Site coordinates.

Site	River	Longitude (°E)	Latitude (°N)
A1	Donghe River	117.549591	29.5229444
A2	Donghe River	117.4032068	29.4590674
A3	Donghe River	117.2856509	29.361594
B1	Chang River	117.2005069	29.3040643
B2	Chang River	117.1552778	29.2447222
B3	Chang River	117.1051168	29.2004044
B4	Chang River	117.0308732	29.1918066
C1	Le'an River	117.43955612	28.9174053
C2	Le'an River	117.1099019	28.9472461
C3	Le'an River	116.99903011	28.8791012

Table S8 (continued). Approximate great-circle distances between sites (km); these are not along-channel distances.

Site	A1	A2	A3	B1	B2	B3	B4	C1	C2	C3
A1	0.0	15.8	31.2	41.7	49.2	56.1	62.3	68.2	76.9	89.3
A2	15.8	0.0	15.7	26.1	33.8	40.8	46.8	60.3	63.6	75.5
A3	31.2	15.7	0.0	10.4	18.1	25.1	31.1	51.6	49.1	60.4
B1	41.7	26.1	10.4	0.0	7.9	14.8	20.7	48.9	40.6	51.1
B2	49.2	33.8	18.1	7.9	0.0	6.9	13.4	45.7	33.4	43.4

Site	A1	A2	A3	B1	B2	B3	B4	C1	C2	C3
B3	56.1	40.8	25.1	14.8	6.9	0.0	7.3	45.2	28.2	37.2
B4	62.3	46.8	31.1	20.7	13.4	7.3	0.0	50.1	28.3	34.9
C1	68.2	60.3	51.6	48.9	45.7	45.2	50.1	0.0	32.3	43.1
C2	76.9	63.6	49.1	40.6	33.4	28.2	28.3	32.3	0.0	13.2
C3	89.3	75.5	60.4	51.1	43.4	37.2	34.9	43.1	13.2	0.0

Table S8 (continued). Environmental measurement methods and available analytical detection limits. Detection limits use the units shown; instrument resolution is not treated as an analytical detection limit.

Variable	Unit	Method	Detection limit
Water temperature	°C	YSI ProQuatro	Not specified
pH	—	YSI ProQuatro	Not specified
Dissolved oxygen	mg/L	YSI ProQuatro	Not specified
Transparency	m	Secchi disk	Not applicable
Total nitrogen	mg/L	Alkaline persulfate digestion–UV spectrophotometry	0.05
Total phosphorus	mg/L	Ammonium molybdate spectrophotometry	0.01
Ammonia nitrogen	mg/L	Nessler spectrophotometry	0.025
Permanganate index	mg/L	Acidic titration with sodium oxalate	0.5
Petroleum	mg/L	Infrared spectrophotometry	0.04
Volatile phenol	mg/L	4-aminoantipyrine spectrophotometry	0.0003
Copper	mg/L	Flameless atomic absorption spectrophotometry	0.01
Chlorophyll *a*	μg/L	Ethanol extraction and spectrophotometry	0.5

Table S8 (continued). Environmental data for the eight retained variables.

Campaign	Site	Temp. (°C)	pH	DO (mg/L)	TN (mg/L)	TP (mg/L)	Phenol (mg/L)	Secchi (m)	Chl a (μg/L)
2023	A1	23.2	7.06	8.36	0.67	0.05	3e-04	2.3	8
2023	A2	22.3	7.2	8.7	0.5	0.03	3e-04	1.92	9
2023	A3	23.9	7.23	8.56	0.51	0.01	3e-04	2.93	10
2023	B1	23.1	6.56	8.66	1.43	0.01	0.002	1.94	12
2023	B2	23.2	7.66	7.62	1.75	0.09	0.0013	2.34	8
2023	B3	23.2	7.53	8.91	1.05	0.05	3e-04	1.56	10
2023	B4	23.4	7.3	8.55	1.68	0.08	3e-04	1.15	8
2023	C1	26	7.95	10.06	0.35	0.05	5e-04	0.81	0
2023	C2	23	7.69	12.34	0.04	0.04	3e-04	1.1	0
2023	C3	25.6	7.67	12.33	0.32	0.06	6e-04	1.35	0
2024	A1	21.9	7.58	6.84	0.6	0.03	0	1.85	3
2024	A2	27.6	7.97	6.75	0.99	0.05	0	1.65	2
2024	A3	27.2	7.7	6.61	0.76	0.03	0	1.21	0
2024	B1	25.7	7.6	6.06	0.91	0.02	0	1.38	0
2024	B2	25	7.37	5.51	1.5	0.04	0	1.51	3
2024	B3	23.9	7.14	6.2	1.33	0.04	0	1.25	2
2024	B4	26.3	7.49	6.23	1.04	0.05	0	0.8	4
2024	C1	27.4	7.88	6.05	1.13	0.05	0	2.23	3
2024	C2	27.9	7.48	6.33	1.16	0.02	0	1.45	3

Campaign	Site	Temp. (°C)	pH	DO (mg/L)	TN (mg/L)	TP (mg/L)	Phenol (mg/L)	Secchi (m)	Chl a (μg/L)
2024	C3	28.8	7.78	6.86	1.43	0.03	0	0.95	3
